# Supplementary material for: Deregulated Expression of Mammalian lncRNA through Loss of SPT6 Induces R-Loop Formation, Replication Stress, and Cellular Senescence
Source: Mol Cell. 2018 Dec 20;72(6):970–984.e7. doi: 10.1016/j.molcel.2018.10.011 (PMC6309921; doi:10.1016/j.molcel.2018.10.011)
Supplement: Document S1. Figures S1–S6 [file mmc1.pdf]

Molecular Cell, Volume *72*

Supplemental Information

Deregulated Expression of Mammalian lncRNA through Loss of SPT6 Induces R-Loop Formation, Replication Stress, and Cellular Senescence

Takayuki Nojima, Michael Tellier, Jonathan Foxwell, Claudia Ribeiro de Almeida, Sue Mei Tan-Wong, Somdutta Dhir, Gwendal Dujardin, Ashish Dhir, Shona Murphy, and Nick J. Proudfoot

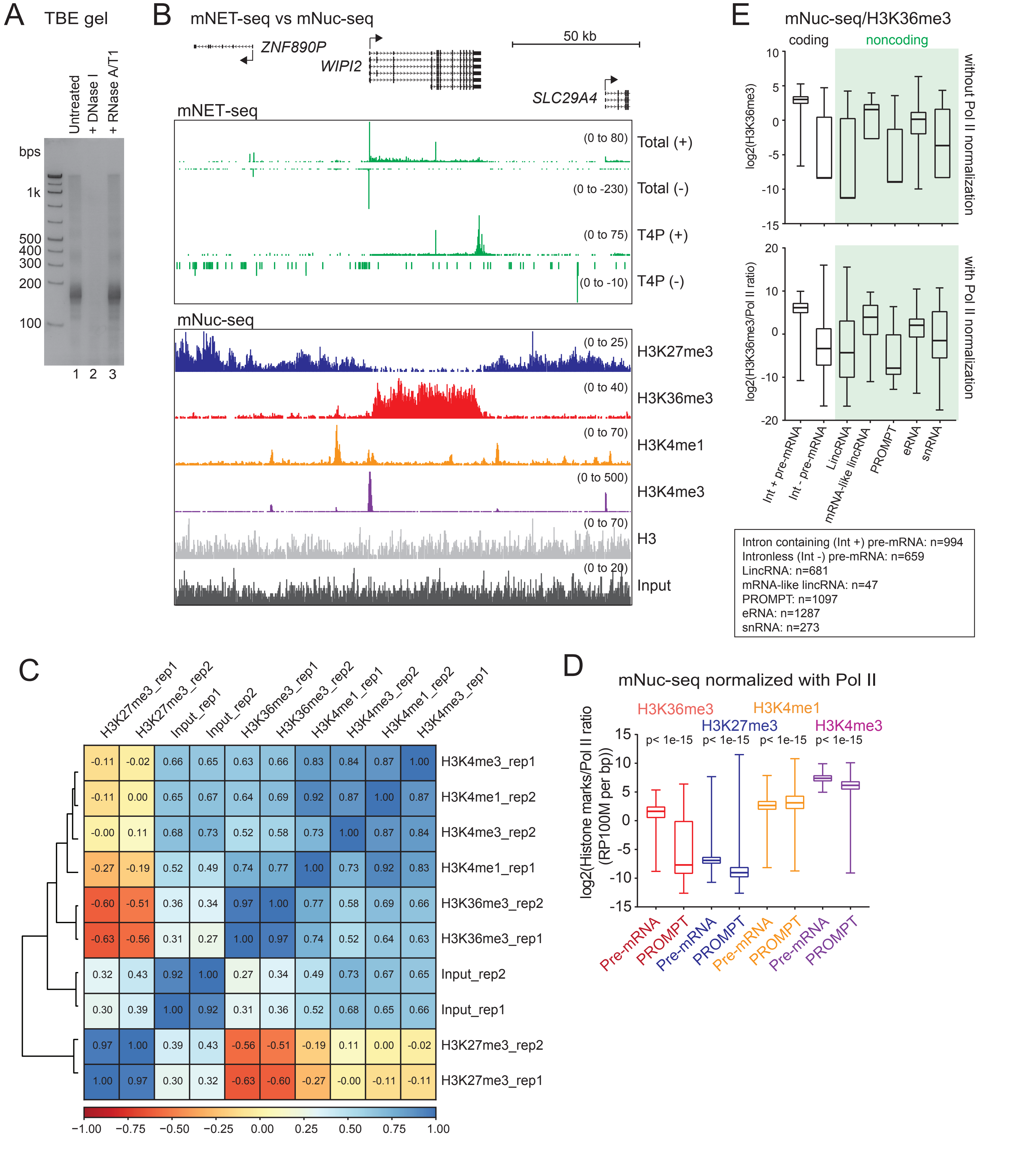

Figure S1. Establishment of mNuc-seq methodology. (related to Figure 1)
(A) Gel image of nucleosome pattern. Following MNase digestion, purified chromatin from untreated (lane 1), DNase I (lane 2) and RNase A/T1 mix (lane 3). Mononucleosome sized (160-180 bps) DNA is predominant.
(B) Wider view (<150 kb) of WIPI2 analysed by mNET-seq/Total and T4P. mNuc-seq/H3K36me3 and K27me3 marks correlate positively and negatively with mNET-seq signals respectively.
(C) Correlation matrix heatmap for mNuc-seq. Positive (+ blue): Negative (- red) showing negative correlation of H3K36me3 and H3K27me3.
(D) Box plots of indicated mNuc-seq signals normalized with mNET-seq/Total CTD signals on pre-mRNA and PROMPT regions.
(E) Box plots of mNuc-seq/H3K36me3 signals normalized without (top) and with (bottom) mNET-seq/Total signals on indicated gene classes.

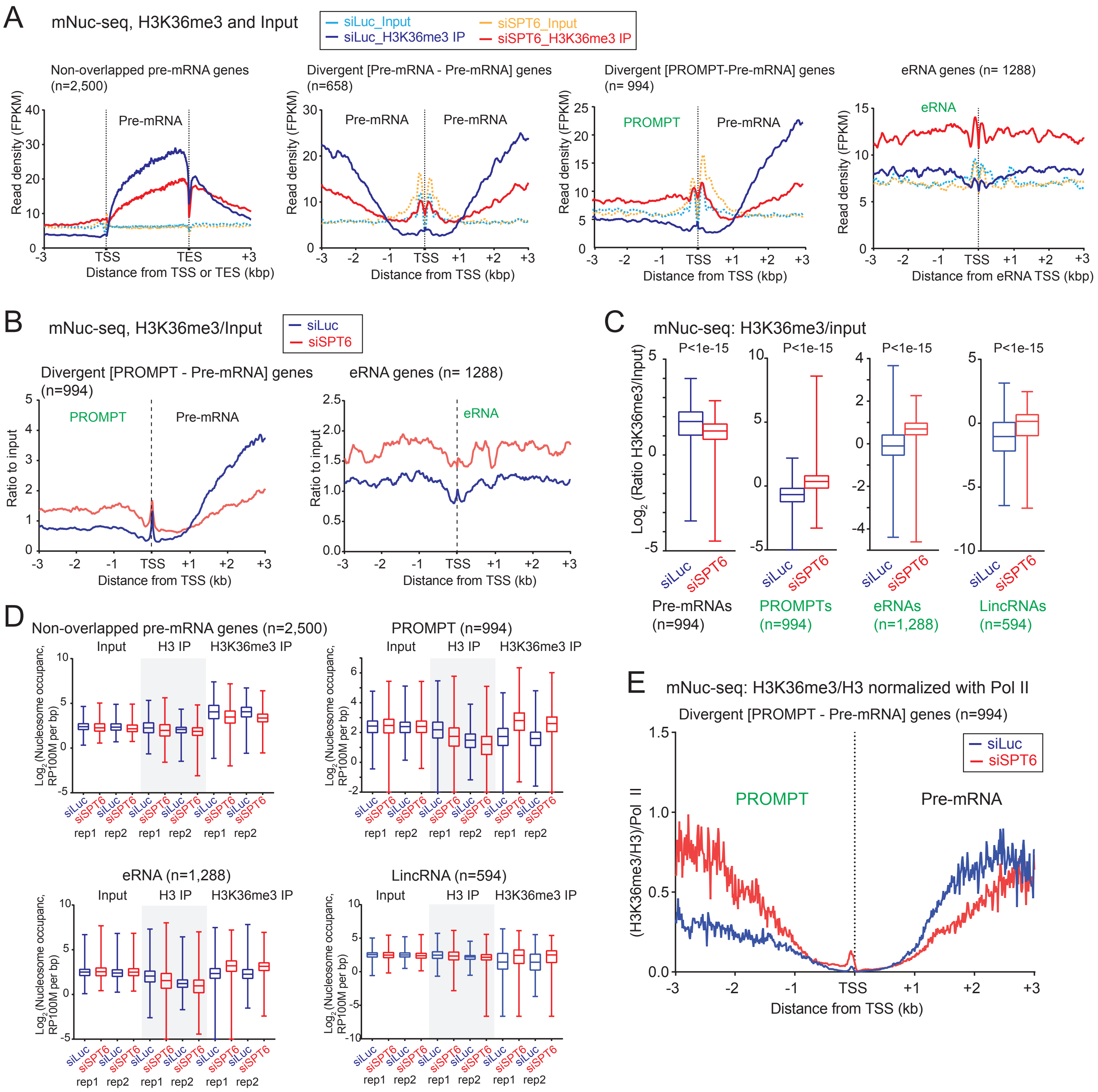

Figure S2. H3K36me3 distribution on pre-mRNA and lncRNA genes. (related to Figure 2)

(A) Meta-analysis of mNuc-seq/H3K36me3 signals over -/+3 kb regions of non-overlapped pre-mRNA genes (left), divergent TUs [Pre-mRNA–Pre-mRNA] (middle left), [PROMPT–Pre-mRNA pairs] (middle right) and enhancers [eRNA-eRNA] (right) upon SPT6 depletion. Dashed lines represent input signals.

(B) Meta-analysis of mNuc-seq/H3K36me3 signal ratioed to input over -/+ 3 kb regions of divergent TUs [PROMPT – Pre-mRNA] (left) and [eRNA -eRNA] (right).

(C) Box plots quantification of mNuc-seq/H3K36me3 ratioed to input on pre-mRNA genes (whole annotated gene), PROMPTs (3 kb from TSS), eRNA (2 kb from the center) and lincRNA (whole annotated gene).

(D) Box plots quantification of absolute signal intensity of mNuc-seq/input, H3 and H3K36me3 on non-overlapping pre-mRNA genes (whole annotated gene), PROMPT (3 kb from TSS), eRNA (2 kb from center) and lincRNA (whole annotated gene) regions. Two replicates are shown.

(E) Meta-analysis of mNuc-seq/H3K36me3 ratioed to H3 and also normalized with Total CTD mNET-seq level over -/+ 3 kb regions of divergent TUs [PROMPT - Pre-mRNA].

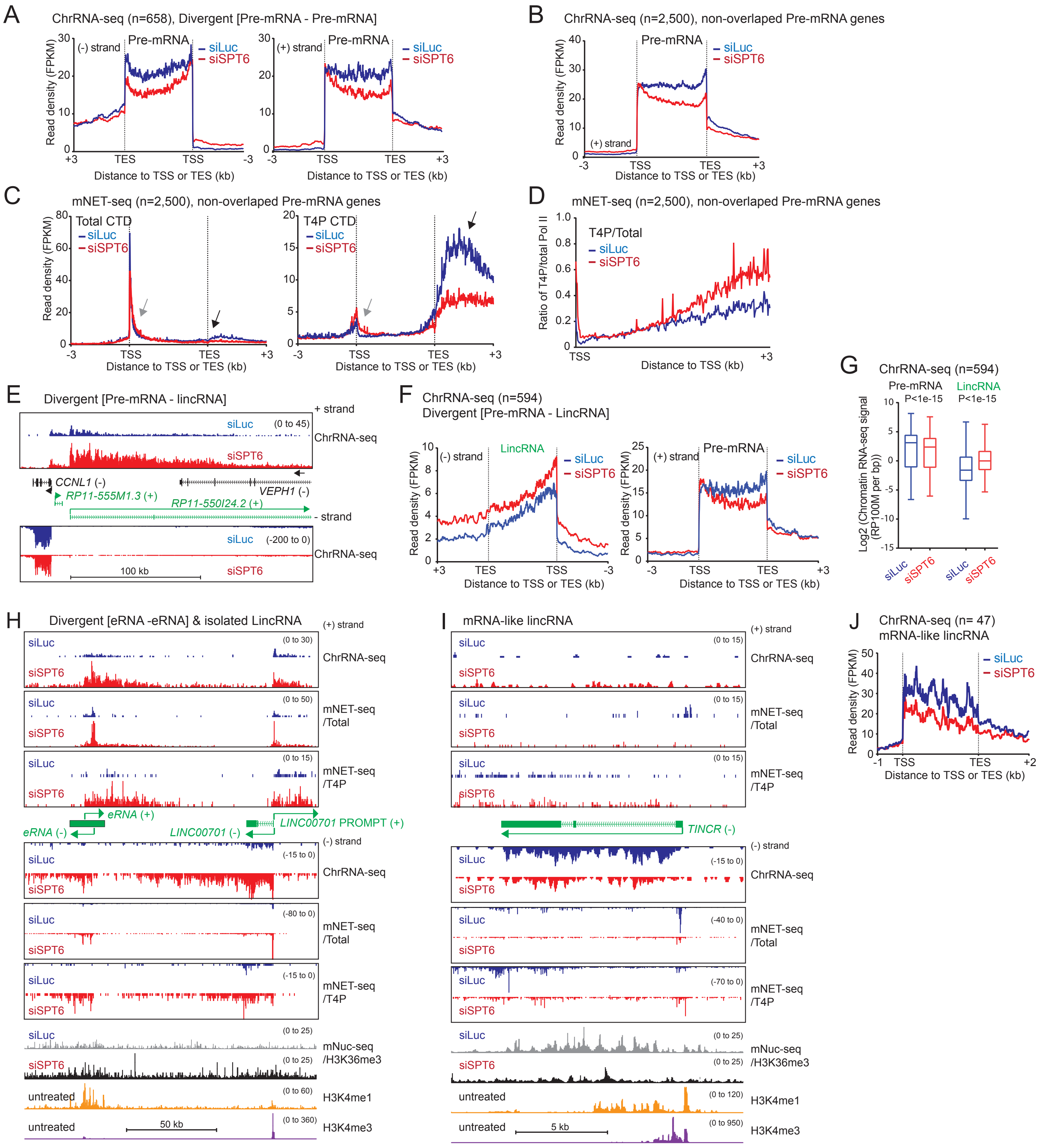

Figure S3. SPT6 depletion reduces Pol II elongation on pre-mRNA, while increasing transcription of lincRNA TUs. (related to Figure 3)
(A) Meta-analysis of reads density for strand-specific ChrRNA-seq signals from 3 kb TSS upstream to 3 kb TES downstream of divergent [Pre-mRNA-Pre-mRNA] genes.
(B) Meta-analysis of reads density for strand-specific ChrRNA-seq signals from 3 kb TSS upstream to 3 kb TES downstream of non-overlapped pre-mRNA genes.
(C) Meta-analysis of reads density for mNET-seq/Total CTD (left) and T4P (right) signals from 3 kb TSS upstream to 3 kb TES downstream of non-overlapped pre-mRNA genes.
(D) Meta-analysis of ratio of mNET-seq/T4P to total CTD from TSS to + 3 kb of non-overlapped pre-mRNA genes.
(E) *CCNL1* and *RP11-550124.2* as example of promoter-associated lincRNA affected by SPT6 depletion.
(F) Meta-analysis of reads density for strand-specific ChrRNA-seq signals from 3 kb TSS upstream to 3 kb TES downstream of divergent [LincRNA - Pre-mRNA] genes.
(G) Box plots of ChrRNA-seq signals (whole annotated gene) of Pre-mRNA and lincRNA pairs [LincRNA - Pre-mRNA].
(H) Examples of eRNA–eRNA pair and linked lincRNA, *LINC00701*.
(I) Example of mRNA-like lincRNA, *TINCR*. mNuc-seq/H3K4me3 and me1 are shown as marks of active promoters and enhancers, respectively.
ChrRNA-seq and mNET-seq profiles (+ and – strands) of *TINCR* show that SPT6 depletion causes transcription elongation defect as for pre-mRNA gene.
(J) Meta-analysis of reads density for strand-specific ChrRNA-seq signals from 3 kb TSS upstream to 3 kb TES downstream of mRNA-like lincRNA TUs upon SPT6 depletion.

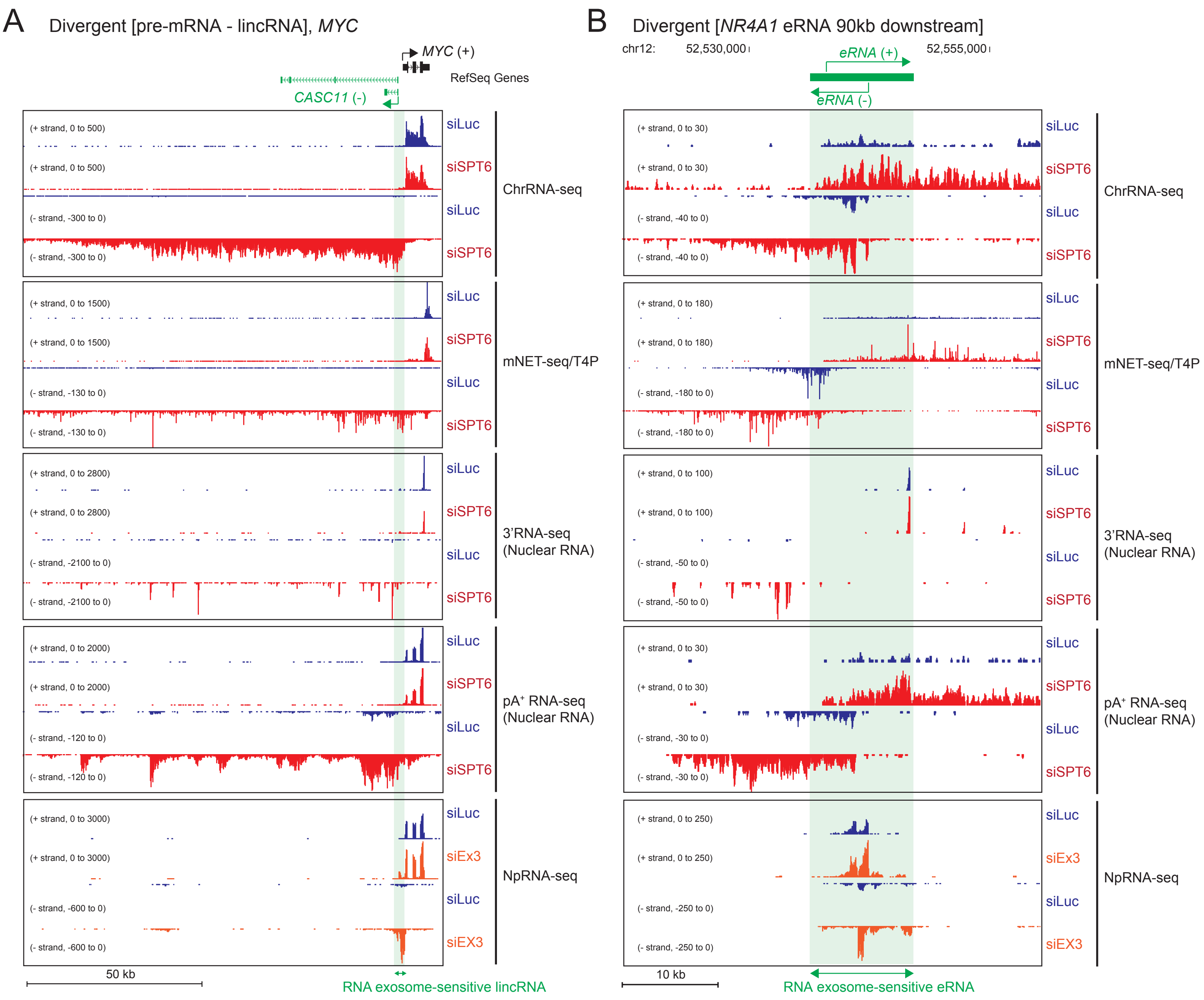

Figure S4. *CASC11* lincRNA - *MYC* pre-mRNA and *NR4A1* eRNA. (related to Figure 4)
(A) Divergent gene [*CASC11* lincRNA-*MYC* pre-mRNA] and (B) Divergent eRNA [*NR4A1* eRNA, 90kb downstream].
From top, strand-specific ChrRNA-seq, mNET-seq/T4P, 3' RNA-seq and pA+ RNA-seq from siLuc and siSPT6 transfected HeLa cells are shown.
NpRNA-seq from siLuc and siEX3 transfected HeLa cells are shown at bottom. RNA exosome-sensitive *MYC* PROMPT is highlighted in green.
SPT6 depletion caused overexpressed and extended lincRNA *CASC11* in chromatin. PAS usage (3'RNA-seq) and increased pA+ signals (pA+RNA-seq) were detected further downstream of highlighted PROMPT region. eRNA stabilised by exosome depletion highlighted in green. SPT6 depletion caused extended eRNA formation.

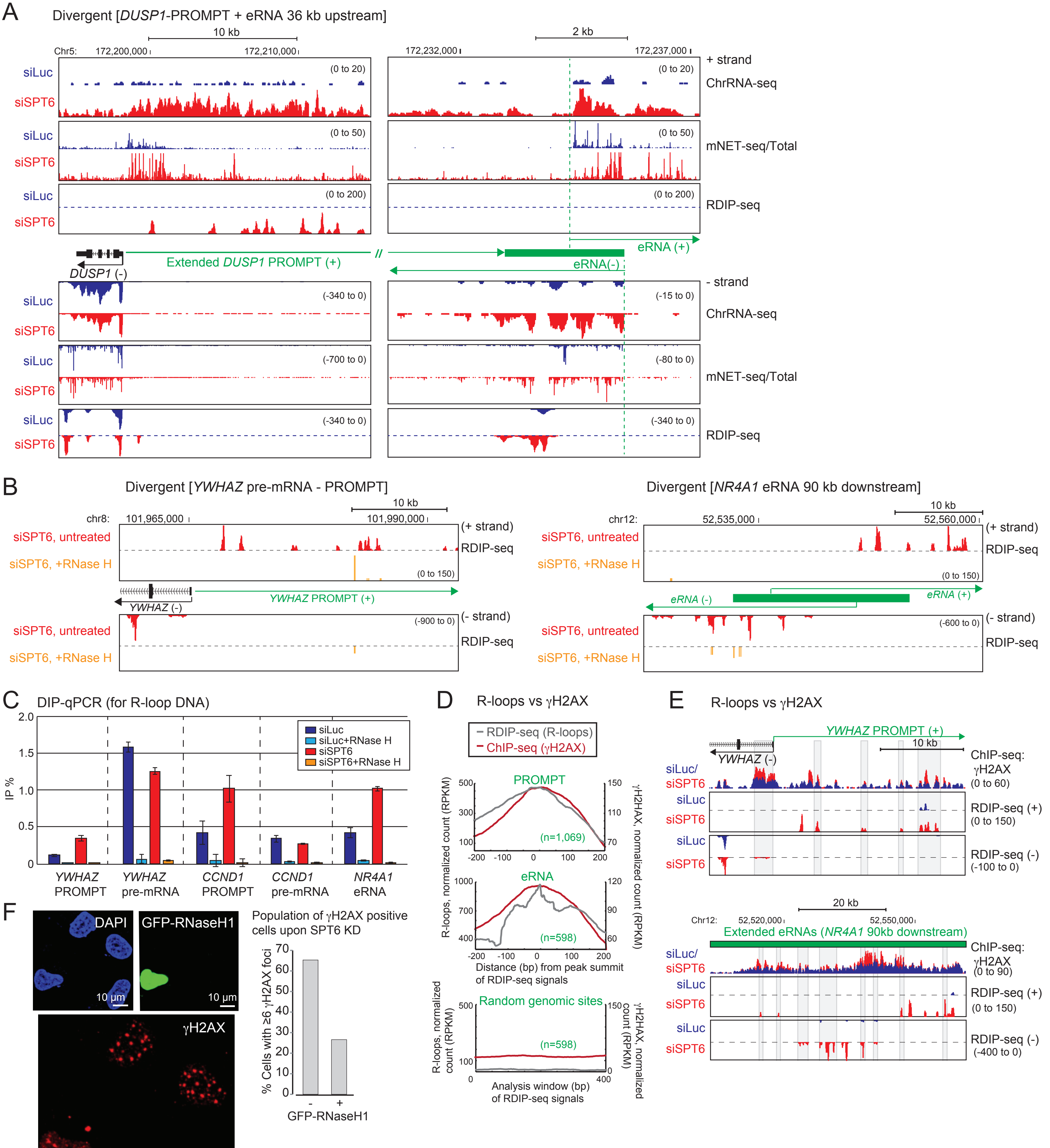

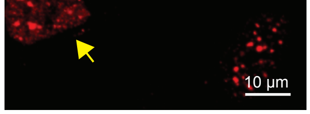

Figure S5. SPT6 depletion induced R-loop associated DNA damage. (related to Figure 6)
(A) *DUSP1* pre-mRNA gene (- strand, left), PROMPT (+ strand, left) and linked eRNA (- and + strands, right) showing R-loop profiles (RDIP-seq) compared to ChrRNA-seq and mNET-seq/Total CTD following SPT6 depletion. Note there is a 20 kb gap between left and right panels.
(B) Specificity of RDIP-seq signals over *YWHAZ* PROMPT and *NR4A1* eRNA regions. Before IP with S9.6 antibody, the IP products were treated with RNase H. In general, significant RDIP-seq peaks were substantially reduced by RNase H treatment.
(C) Quantitative DIP-PCR of indicated candidate gene loci. SPT6 depletion increased DIP signals in PROMPTs and eRNA, but decreased signal in pre-mRNA. In vitro RNase H treatment significantly reduced all DIP signals. Data is represented as mean ± SEM.
(D) Meta-analysis of RDIP-seq and γH2AX ChIP-seq in SPT6-depeleted HeLa cells for PROMPT and eRNA R-loop peak regions. Distance shown from RDIP-seq peak summit. Random genomic regions (400bp window) are shown as control.
(E) *YWHAZ* PROMPT and *NR4A1* eRNA are shown as examples of the peak summit analyses in (D). γH2AX ChIP-seq signals were overlapped in siLuc (blue) and siSPT6 (red) samples. R-loop peaks with increased γH2AX ChIP-seq signals are highlighted in grey.
(F) Immunofluorescence analysis of γH2AX after GFP-RNase H1 over-expression. A field of 4 cells is shown with 1 cell over-expressing RNase H1. γH2AX IF is 2-fold enlarged. Note that the RNase H1 over-expressing cell shows fewer foci. Cells which contains >6 nuclear foci of γH2AX were counted upon SPT6 knockdown.

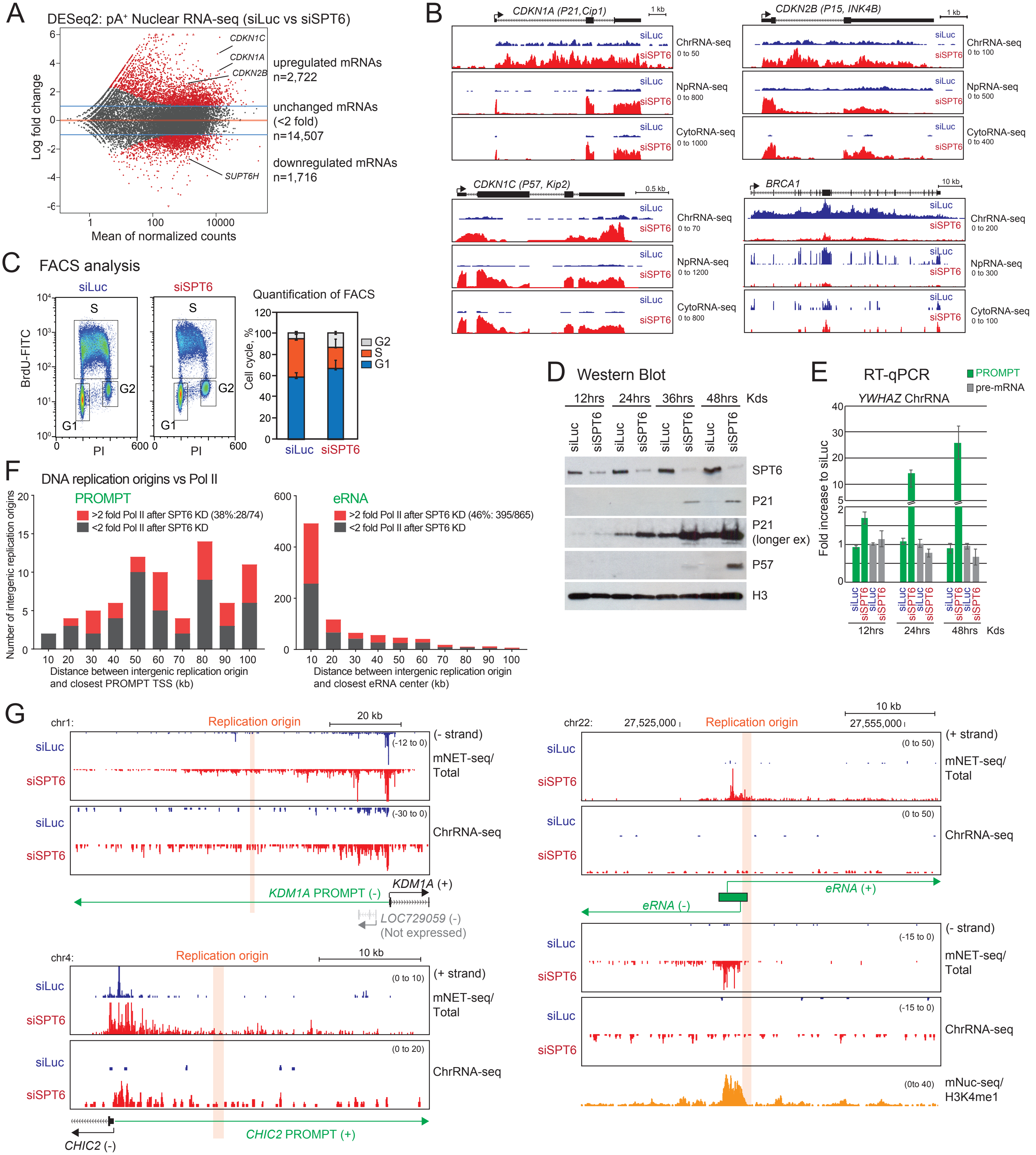

Figure S6. SPT6 depletion causes collision between Pol II and DNA replisome. (related to Figure 7)

(A) Differential expression-analysis (DESeq2) of nuclear pA$^+$ RNA-seq signals from HeLa nucleus. >2-fold upregulated, > 2-fold downregulated and unchanged (<2-fold). Expression of cell cycle inhibitor genes such as *CDKN1A*, *CDKN1C* and *CDKN2B* were significantly upregulated while siRNA-target *SUPT6H* was downregulated.

(B) Examples of siSPT6-affected gene expression in different HeLa cell fractions: chromatin (ChrRNA-seq), nucleoplasm (NpRNA-seq) and cytoplasm (CytoRNA-seq). Signals of cell cycle inhibitor genes, *CDKN1A*, *CDKN1C* and *CDKN2B* were upregulated in all three fractions. In contrast DNA damage response gene *BRAC1* was down-regulated after SPT6 siRNA depletion.

(C) Cell cycle analysis by fluorescence activated cell sorting (FACS) of HeLa cells following SPT6 depletion. Signals were quantified from three biological replicates of SPT6-depleted HeLa cells. Data is represented as mean ± SEM.

(D) The whole HeLa cell extracts were prepared after 12, 24, 36 and 48 hr control and SPT6 siRNAs transfection for western blot against indicated antibodies. P21 levels are shown with short and long exposure. Note P21 protein level was upregulated after 24 hr SPT6 depletion.

(E) Quantitative RT-PCR of chromatin-bound RNA. *YWHAZ* PROMPT and pre-mRNA were analyzed after 12, 24 and 48 hr control and SPT6 siRNAs transfection. Data are represented as mean ± SEM.

(F) Spatial relationship between Pol II and DNA replication origin in intergenic region. Replication origins with elevated Pol II level (>2 fold) in SPT6 depleted cells are highlighted in red.

(G) Three examples of mNET-seq/Total profiles in intergenic DNA replication origins.
