# Supplementary material for: Deregulated Expression of Mammalian lncRNA through Loss of SPT6 Induces R-Loop Formation, Replication Stress, and Cellular Senescence
Source: Mol Cell. 2018 Dec 20;72(6):970–984.e7. doi: 10.1016/j.molcel.2018.10.011 (PMC6309921; doi:10.1016/j.molcel.2018.10.011)
Supplement: Document S2. Article plus Supplemental Information [file mmc4.pdf]

Molecular Cell

Article

Deregulated Expression of Mammalian lncRNA through Loss of SPT6 Induces R-Loop Formation, Replication Stress, and Cellular Senescence

Graphical Abstract

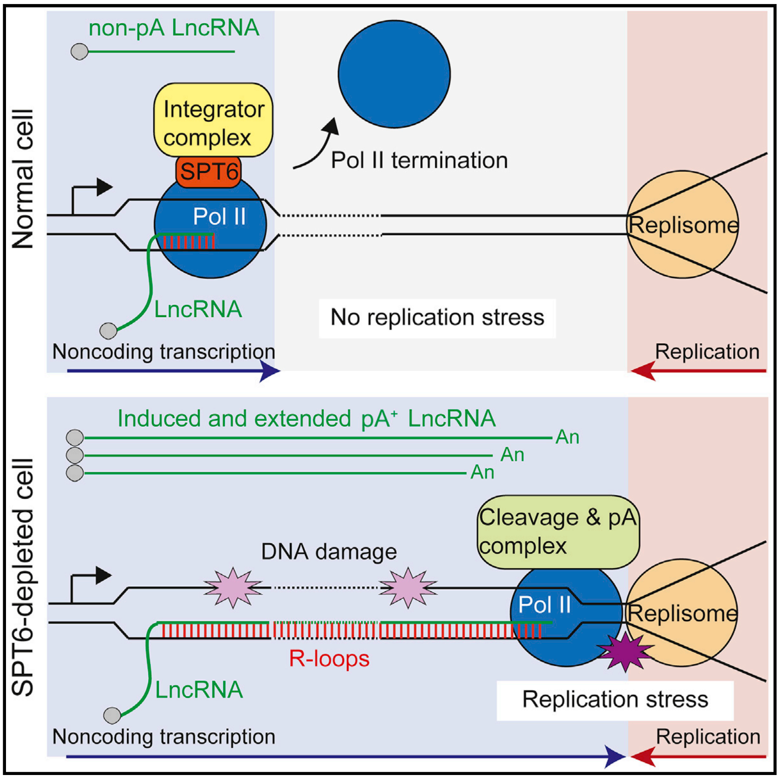

Authors

Takayuki Nojima, Michael Tellier, Jonathan Foxwell, ..., Ashish Dhir, Shona Murphy, Nick J. Proudfoot

Correspondence

taka.nojima@path.ox.ac.uk (T.N.),
shona.murphy@path.ox.ac.uk (S.M.),
nicholas.proudfoot@path.ox.ac.uk (N.J.P.)

In Brief

Nojima and colleagues demonstrate that loss of human SPT6 protein elevates levels of noncoding transcription and perturbs its termination. The deregulated noncoding transcription increases R-loop formation with consequent DNA damage, transcription-replication collision, and cellular senescence. This implies that noncoding transcription is restricted to avoid such conflicts in proliferating cells.

Highlights

- SPT6 promotes the selective distribution of H3K36me3 over protein-coding genes
- SPT6 loss leads to formation of extended lncRNAs that are prone to R-loop formation
- Deregulated Pol II collides with DNA replisomes on lncRNA genes
- Collision between Pol II and DNA replisome leads to cellular senescence

Nojima et al., 2018, Molecular Cell *72*, 970–984
December 20, 2018 © 2018 The Authors. Published by Elsevier Inc.
https://doi.org/10.1016/j.molcel.2018.10.011

CellPress
Molecular Cell
Article

Deregulated Expression of Mammalian lncRNA through Loss of SPT6 Induces R-Loop Formation, Replication Stress, and Cellular Senescence

Takayuki Nojima,[1,2,3,*] Michael Tellier,[1,2] Jonathan Foxwell,[1] Claudia Ribeiro de Almeida,[1] Sue Mei Tan-Wong,[1] Somdutta Dhir,[1] Gwendal Dujardin,[1] Ashish Dhir,[1] Shona Murphy,[1,3,*] and Nick J. Proudfoot[1,3,4,*]
[1]Sir William Dunn School of Pathology, University of Oxford, South Parks Road, Oxford OX1 3RE, UK
[2]These authors contributed equally
[3]Senior author
[4]Lead Contact
*Correspondence: taka.nojima@path.ox.ac.uk (T.N.), shona.murphy@path.ox.ac.uk (S.M.), nicholas.proudfoot@path.ox.ac.uk (N.J.P.)
https://doi.org/10.1016/j.molcel.2018.10.011

SUMMARY

Extensive tracts of the mammalian genome that lack protein-coding function are still transcribed into long noncoding RNA. While these lncRNAs are generally short lived, length restricted, and non-polyadenylated, how their expression is distinguished from protein-coding genes remains enigmatic. Surprisingly, depletion of the ubiquitous Pol-II-associated transcription elongation factor SPT6 promotes a redistribution of H3K36me3 histone marks from active protein coding to lncRNA genes, which correlates with increased lncRNA transcription. SPT6 knockdown also impairs the recruitment of the Integrator complex to chromatin, which results in a transcriptional termination defect for lncRNA genes. This leads to the formation of extended, polyadenylated lncRNAs that are both chromatin restricted and form increased levels of RNA:DNA hybrid (R-loops) that are associated with DNA damage. Additionally, these deregulated lncRNAs overlap with DNA replication origins leading to localized DNA replication stress and a cellular senescence phenotype. Overall, our results underline the importance of restricting lncRNA expression.

INTRODUCTION

RNA polymerase II (Pol II) transcribes much of the eukaryotic genome, dividing its activity between protein-coding genes and several classes of long noncoding RNA (lncRNA). These include long intergenic noncoding RNA (lincRNA), antisense promoter upstream transcripts (PROMPTs), and bidirectional enhancer-associated transcripts or enhancer RNA (eRNA) (St Laurent et al., 2015). Both lncRNA and pre-mRNA transcription units (TUs) are subject to co-transcriptional processing by 5′ capping enzymes, the spliceosome, and the 3′ cleavage and polyadenylation (CPA) complex. However, splicing and CPA are inefficient for lncRNAs (Mukherjee et al., 2017; Schlackow et al., 2017) leading to their rapid degradation by the nuclear exosome. In effect, functional lncRNA must escape this degradation process. Possibly tissue-specific RNA binding factors act to protect and stabilize functional lncRNA (Schlackow et al., 2017). The reduced efficiency of lncRNA co-transcriptional processing likely correlates with reduced phosphorylation of elongating Pol II on its C-terminal domain (CTD) especially the splicing associated S5P and 3′ end-associated T4P modifications.

Some classes of lncRNA such as PROMPTs are transcribed from bidirectional promoters of protein-coding genes, although not all Pol II promoter regions display such bidirectionality. Several mechanisms act to restrict divergent transcription. In general, well-defined upstream *cis*-elements of promoters such as the TATA box provide an important determinant of unidirectional promoters. Notably, CpG-rich promoters that lack TATA boxes are often a feature of bidirectional promoters in the mammalian genome (Sandelin et al., 2007). Also, nucleosome remodeling and associated alterations in histone modification can restrict antisense transcription (Marquardt et al., 2014; Whitehouse et al., 2007). Another numerous class of divergent transcription derives from transcriptional enhancer elements (Li et al., 2016). In view of the abundance and tissue specificity of enhancers, including clustered, or so-called super enhancers, eRNAs are both widespread and often cell type specific (Li et al., 2016). As with other lncRNA classes, eRNAs are inefficiently spliced and in general lack consensus splicing signals (Andersson et al., 2014).

The Pol II termination mechanism for lncRNA genes also appears to be distinct from protein-coding genes, even though computational and 3′ RNA sequencing (RNA-seq) analyses do identify functional polyadenylation signals (PASs) on lncRNA genes (Ulitsky and Bartel, 2013). For most lincRNA, nascent transcript analysis as measured by mammalian native elongating transcript sequencing (mNET-seq) implies a PAS-independent termination mechanism (Schlackow et al., 2017). Instead, for PROMPTs, it has been suggested that they are prematurely terminated by cryptic PAS (Almada et al., 2013; Ntini et al., 2013). While such PASs are also present throughout protein-coding genes, these are normally blocked by splicing signals and in particular by U1 small nuclear RNA (snRNA) (Kaida

 © 2018 The Authors. Published by Elsevier Inc.
This is an open access article under the CC BY license (http://creativecommons.org/licenses/by/4.0/).

et al., 2010). Notably, splice sites are more common in pre-mRNA than lncRNA transcripts, which favor full-length pre-mRNA synthesis. However, short promoter-proximal transcripts on the sense strand can still be terminated by premature polyadenylation (Chiu et al., 2018). eRNAs have also been ascribed a specific termination mechanism involving the Integrator complex (Lai et al., 2015). This multimeric protein possesses components with homology to CPA and especially CPSF73, the endonuclease required for mRNA 3′ end processing (Baillat and Wagner, 2015). Likely eRNA termination is also associated with co-transcriptional 3′ end processing, coupled to RNA degradation.

As outlined above, lncRNA gene expression displays specific transcription and processing features that distinguishes it from protein-coding genes. Since both transcript classes are transcribed by Pol II, it is unclear how lncRNAs are selected for a different gene expression outcome to protein-coding genes. Initially, we compared nascent RNA generated from bidirectional promoters and its histone modifications. As expected, the histone mark H3K36me3 is enriched over protein-coding gene TUs, compared to PROMPTs and enhancers. Notably, we find that the elongation factor SPT6, which is required both for escape of Pol II from promoter pausing (Vos et al., 2018) and subsequent elongation (Endoh et al., 2004), plays a key role in defining protein-coding gene TUs and in restricting lncRNA transcription. Thus, depletion of SPT6 activates and extends lncRNA, and this deregulated Pol II leads to molecular collisions with the DNA replisome in intergenic regions. Also, these elevated, aberrant lncRNAs anneal to the DNA template forming R-loop structures that in turn induce DNA damage. Overall, this perturbation of lncRNA expression leads to cell-cycle arrest and senescence, which underlines the critical importance of restricting lncRNA expression.

RESULTS

H3K36me3 Is a Predominant Histone Mark for Protein-Coding Genes

We have combined chromatin-associated RNA sequencing (ChrRNA-seq) with specific Pol II isoform-associated RNA sequencing (mNET-seq) to highlight differences between protein-coding and lncRNA gene expression (Nojima et al., 2015; Schlackow et al., 2017). This led us to hypothesize that the Pol II machinery and its underlying chromatin template differ between these two transcript types. We first examined histone modification profiles by adapting our mNET-seq procedure to directly compare nucleosome profiles (mono nucleosome DNA-sequencing [mNuc-seq]; Figure 1A) with mNET-seq. In mNuc-seq, chromatin DNA fragmented by micrococcal nuclease (MNase) is precipitated by histone-specific antibodies followed by selection of mononucleosome-sized DNA fragments and sequencing (Figure S1A). This provides genome-wide nucleosome profiles that can be exactly correlated with nascent transcript mapping by mNET-seq. Using an H3K4me3 antibody, mNuc-seq produced positioned nucleosome profiles at the transcription start site (TSS) of *PRPF38B* at higher resolution than previously published chromatin immunoprecipitation sequencing (ChIP-seq) profiles (Figure 1B). Note the clear nucleosome-depleted region (NDR) between the TSS of *PRPF38B* and its PROMPT. Meta-analysis of mNET-seq versus mNuc-seq/H3K4me3 shows that Pol II pausing at the TSS positively correlates with nucleosomes at −1 and +1 positions (Figure 1C).

Next, we compared the genomic profiles of mNuc-seq using antibodies specific for H3K27me3, H3K36me3, H3K4me1, and H3K4me3. Analysis of the genes *YWHAZ* (Figure 1D) and in a wider genomic context *WIP12* (Figure S1B) shows a number of predicted patterns of chromatin marks (Bannister and Kouzarides, 2011) that we can now closely correlate with mNET-seq. The *WIPI2* TU is defined at its TSS by a mNET-seq/total Pol II peak and its transcription end site (TES) by high termination-specific mNET-seq/T4P reads. Notably, the H3K36me3 signal is specific for *WIPI2* TU with chromatin outside the TU enriched instead for H3K27me3 marks (Figure S1B). All the mNuc-seq libraries described in this study were reproduced and show clear anti-correlation between H3K36me3 and H3K27me3 (Figure S1C). H3K4me1 and H3K4me3 chromatin marks are well known to correlate with poised enhancers and active promoters respectively (Calo and Wysocka, 2013). Across the *YWHAZ* and *WIP12* loci, we observe H3K4me3 at promoters, while H3K4me1 chromatin mark appears less specific but may correlate with potential enhancer regions (Figures 1D and S1B). We also carried out statistical analysis of these various histone marks with or without Pol II (mNET-seq) normalization across the HeLa cell genome (Figures 1E and S1D). Notably, H3K36me3 is exclusive to protein-coding genes with introns and mRNA-like lincRNA but absent or at low levels on protein-coding genes without introns and all other categories of lncRNA (Figure S1E). Overall, this high-resolution methodology for the correlation of histone marks with nascent transcription (i.e., comparing mNET-seq with mNuc-seq) underlines the exclusive presence of H3K36me3 marks over most protein-coding but not lncRNA TUs. This led us to investigate whether this mark defines the difference between protein-coding and lncRNA gene expression.

SPT6 Selectively Recruits H3K36me3 to Protein-Coding Genes

We have recently adapted our mNET-seq technique, which sequences Pol II immunoprecipitated (IPed) RNA to develop mNET-MS analysis that identifies (by mass spectroscopy or MS) proteins interacting with each phospho-CTD-specific Pol II isoform. Using this method, we showed that the phosphoserine 5 (S5P) CTD isoform of Pol II specifically recruits the catalytic spliceosome (Nojima et al., 2018). Interestingly, we also detected SPT6 as strongly associated with Pol II, independent of the CTD phosphorylation state. Yeast SPT6 has been shown to interact with histone H3 (Bortvin and Winston, 1996) and also with Pol II machinery through association of its SH2 domain with the Pol II Rpb1 linker sequence that connects the CTD to the main globular enzyme (Sdano et al., 2017). This later association has been reaffirmed by cryoelectron microscopy (cryo-EM) analysis of *in vitro* reconstituted early elongation complexes (Vos et al., 2018). Importantly, SPT6 has also been strongly implicated in the deposition of H3K36me3 in yeast (DeGennaro et al., 2013) and interacts with IWS1, which associates with H3K36 methyltransferase SETD2 in human cells (Yoh et al., 2008).

To confirm the role of human SPT6 in maintaining H3K36me3 marks over protein-coding genes, we depleted SPT6 protein

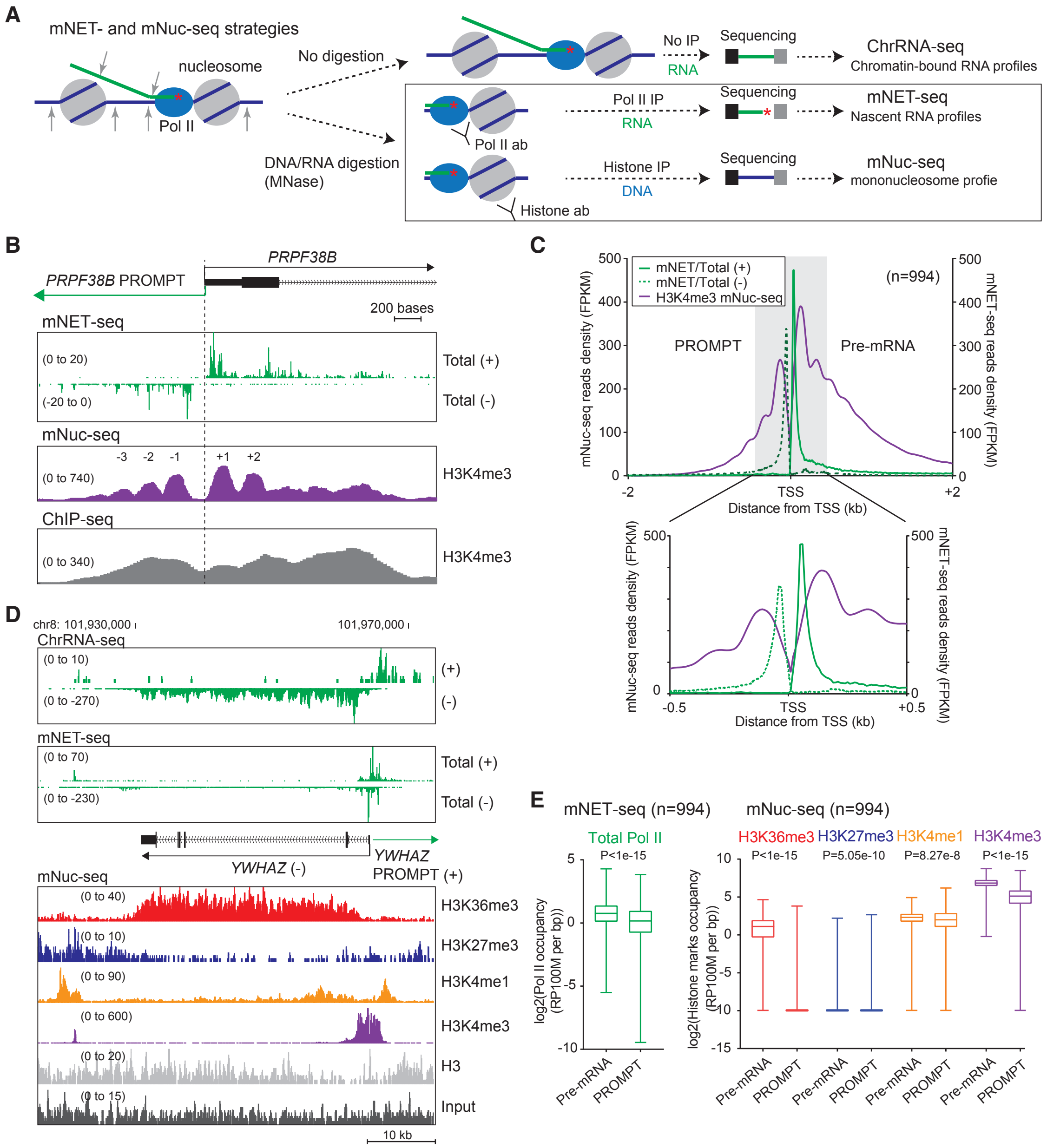

Figure 1. mNuc-seq Identifies H3K36me3 as a Predominant Mark for Protein-Coding Genes

(A) ChrRNA-seq, mNET-seq, and mNuc-seq methodology. Nucleic acid, protected by the Pol II complex and mononucleosomes, is immunoprecipitated by Pol II or histone antibodies followed by sequencing.

(B) *PRPF38B* TSS showing mNET-seq/total CTD, mNuc-seq/H3K4me3, and ChIP-seq/H3K4me3 (ENCODE) profiles. Bracketed numbers denote read density (fragments per kilobase of transcript per million mapped reads [FPKM]). TSS-associated nucleosomes are indicated.

(C) Meta-analysis of reads density for mNET-seq/total CTD (green) versus mNuc-seq/H3K4me3 (purple) signals at TSS (top, –/+2 kb; bottom; –/+ 0.5 kb) of pre-mRNA genes.

(D) *YWHAZ* TSS-aligned ChrRNA-seq, mNET-seq/total CTD profiles versus mNuc-seq using indicated histone antibodies. Input signal is at bottom.

(E) Box plots of mNET-seq/total CTD and mNuc-seq signals at TSS (–/+3 kb) of pre-mRNA genes and associated PROMPTs.

See also Figure S1.

levels in HeLa cells using small interfering RNAs (siRNAs) as shown by western blots of both whole-cell and chromatin fractions (Figure 2A). Notably, SPT6 depletion does not affect levels of Pol II CTD S5P or S2P. Next, we performed mNuc-seq for H3K36me3 on protein-coding and lncRNA genes with or without SPT6 depletion (Figures 2B and S2A). As expected following SPT6 depletion, levels of H3K36me3 normalized to H3 (Figure 2B) or input (Figure S2B) specifically decreased on protein-coding genes (labeled Pre-mRNAs) associated with divergent TUs and non-overlapped protein-coding genes. It should be

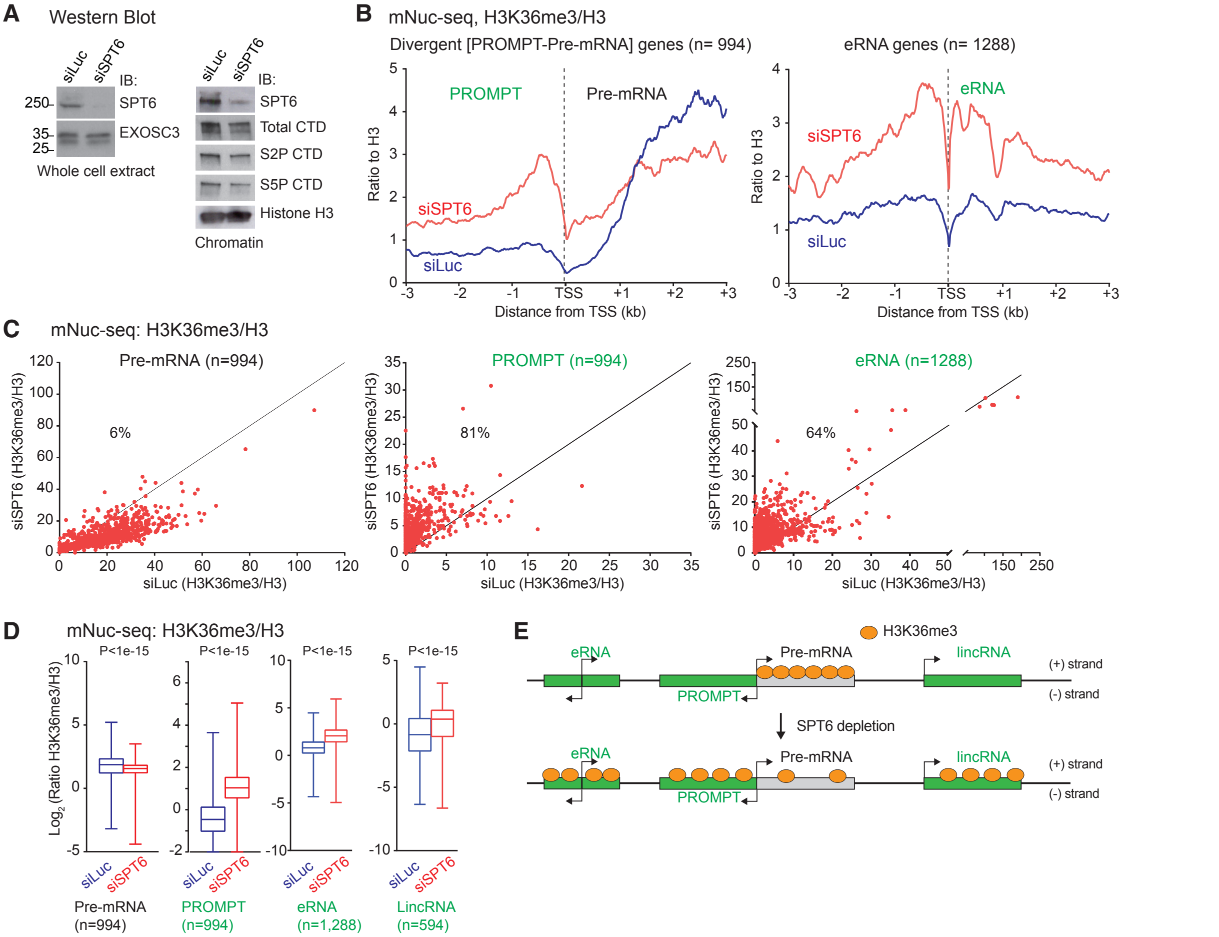

Figure 2. SPT6 Depletion Causes H3K36me3 Redistribution

(A) Western blots with indicated antibodies 60 hr post-siSPT6 transfection (versus siLuc nonspecific control). EXOSC3 and H3 profiles shown as loading controls.

(B) Meta-analysis of reads density (FPKM) for ratio of mNuc-seq/H3K36me3 with H3 at TSS of divergent PROMPT-pre-mRNA and enhancer RNA (eRNA) following SPT6 depletion. All subsequent transcription images employing siSPT6 versus siLuc are shown in red and blue, respectively.

(C) Scatterplots of H3K36me3/H3 on pre-mRNA (0 to +3 kb), PROMPT (−3 kb to 0), and eRNA (−3 kb to +3 kb) regions in siLuc versus siSPT6. The percentage of upregulated regions by SPT6 depletion are indicated.

(D) Boxplots of mNuc-seq/H3K36me3 ratio across pre-mRNA gene bodies, PROMPTs (3 kb from TSS), and eRNA (2 kb from center) and across lincRNA gene bodies.

(E) Model of redistributed H3K36me3 marks caused by SPT6 depletion.

See also Figure S2.

noted that H3 is detectible over TSS regions despite nucleosome depletion. Chromatin is only partially digested by MNase in our mNuc-seq protocol so that histone signals are still detectible over NDRs (Mieczkowski et al., 2016; Voong et al., 2016). Surprisingly SPT6 depletion had the opposite effect on lncRNA such as PROMPTs and eRNA as shown by metagene and scatterplot analysis where H3K36me3 signals increase (Figures 2B and 2C). Quantification of the mNuc-seq signal ratio of H3K36me3 IP to input or H3 also normalized with Pol II levels indicates that SPT6 depletion causes loss of H3K36me3 mark on protein-coding genes, but a gain of this mark on lncRNA genes such as PROMPTs, eRNA, and lincRNA (Figures 2D and S2C–S2E). Overall, our mNuc-seq data show that SPT6 plays a critical role in defining the specificity of H3K36me3 between pre-mRNA and lncRNA genes (Figure 2E).

SPT6 Depletion Induces lncRNA Transcription

We next measured nascent transcript levels with or without SPT6 depletion for both chromatin-bound RNA (ChrRNA) and Pol-II-associated RNA (mNET-seq). For mNET-seq, we used total and phospho Thr4 (T4P) CTD antibodies as the later profiles correlate with transcriptional termination for protein-coding genes but are spread across the whole TU of lincRNA genes (Schlackow et al., 2017). We initially analyzed the transcription profile of *YWHAZ*, a divergent TU (PROMPT-pre-mRNA) (Figure 3A, – strand). Following SPT6 depletion, ChrRNA-seq and mNET-seq signals are reduced near the *YWHAZ* TU 3′ end (TES) indicative of a transcription elongation defect. Meta-analysis of ChrRNA-seq signals for protein-coding genes, including both divergent (pre-mRNA-pre-mRNA) and non-overlapping also show clear elongation defects for SPT6-depleted cells. In

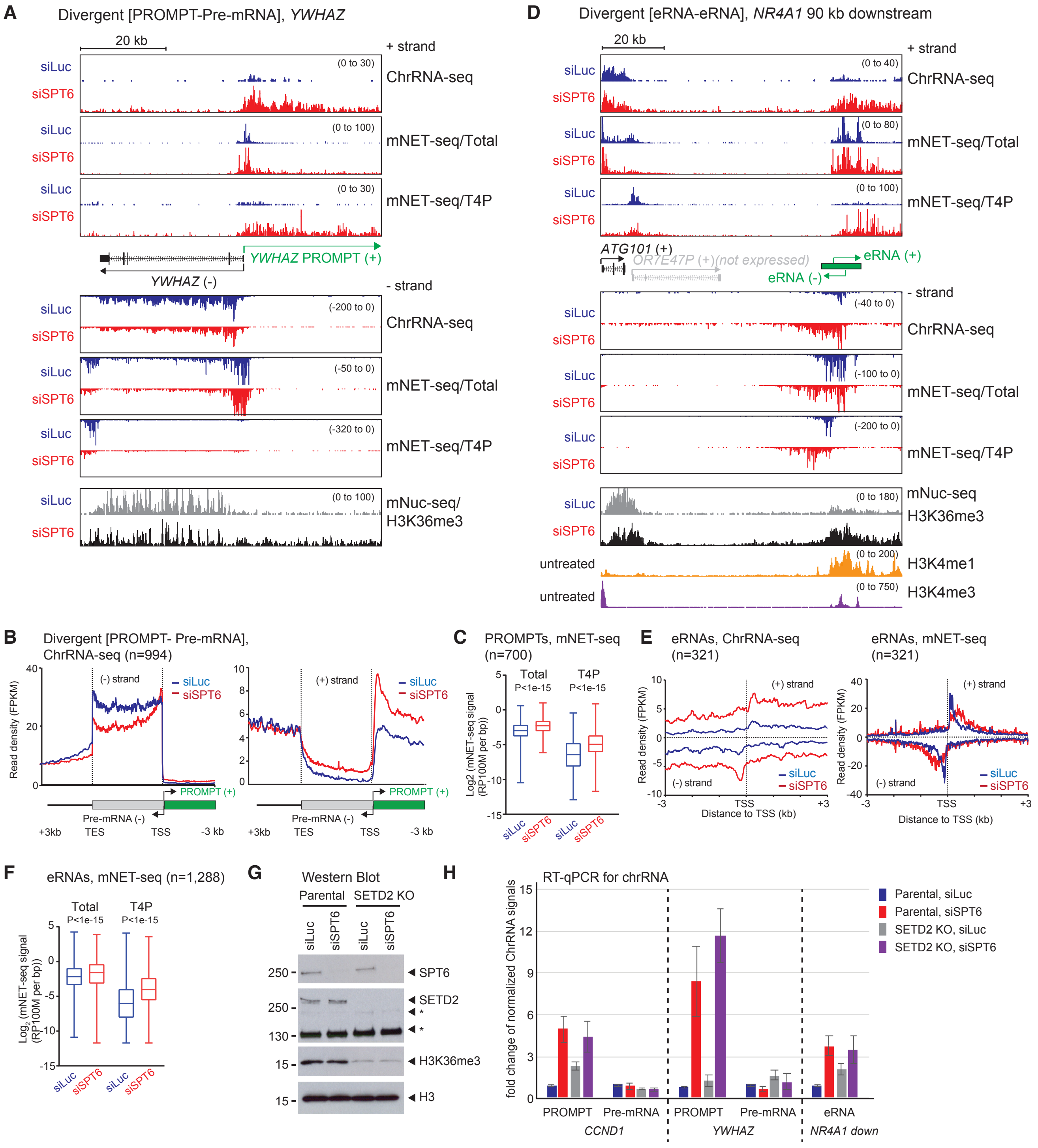

Figure 3. SPT6 Depletion Induces lncRNA Transcription

(A) *YWHAZ* locus showing pre-mRNA (– strand) and PROMPT (+ strand). SPT6 depletion induced lncRNA as shown by ChRNA-seq and mNET-seq analyses. The profile of mNuc-seq/H3K36me3 is shown below.

(B) Meta-analysis of strand-specific ChrRNA-seq signals from −3 kb of TSS to +3 kb of transcription end site (TES) for divergent (pre-mRNA-PROMPT) genes.

(C) Boxplots of PROMPT mNET-seq/total CTD and T4P signals.

(D) Enhancer located 90 kb downstream of *NR4A1* gene with neighboring gene *ATG101* showing SPT6 depletion-induced eRNA by ChRNA-seq and mNET-seq. mNuc-seq/H3K4me1 and H3K4me3 signals indicate active enhancer and promoter, respectively.

(E) Meta-analysis of eRNA from ChrRNA-seq and mNET-seq (Total) −3 kb to +3 kb from TSS (enhancers with highest eRNA levels selected).

(legend continued on next page)

addition, meta-analyses of mNET-seq signals detects more Pol II near TSS, but less from gene body to TES in the depleted cells again consistent with an elongation defect (Figures 3B and S3A–S3C). SPT6 depletion also shows that T4P marks are significantly enriched within 3 kb of the TSSs of protein-coding genes (Figure S3D). These results indicate that the depletion of SPT6 induces transcription elongation defects and premature termination.

An opposite effect is apparent for lncRNA following SPT6 depletion. Thus, levels of *YWHAZ* PROMPT substantially increase following knockdown of SPT6 protein as demonstrated by ChrRNA-seq and mNET-seq profiles (Figure 3A, + strand). Furthermore, meta-profiles of ChrRNA-seq signals (Figure 3B) and the quantification of mNET-seq/total Pol II and T4P signals (Figure 3C) confirm this generality of enhanced lncRNA transcription. Notably, transcription levels of other lncRNA classes such as eRNA and lincRNA similarly increase after SPT6 depletion (Figures 3D–3F and S3E–S3H). In particular, the enhancer sequence 90 kb downstream of *NR4A1* (Figure 3D) is defined by a high H3K4me1 signature (based on mNuc-seq). Bidirectional eRNAs are evident for this enhancer as judged by ChrRNA-seq and mNET-seq (with both total and T4P-specific Pol II antibodies). Notably, with each nascent transcriptional analysis, SPT6 depletion causes a substantial transcript increase in both eRNA orientations. In contrast, the minority mRNA-like lincRNA show decreased transcriptional elongation similarly to pre-mRNA (Figures S3I and S3J). Overall our findings that SPT6 depletion oppositely affects protein-coding and lncRNA genes indicate that this protein plays a pivotal role in defining Pol II TUs across the human genome. Thus, SPT6 favors expression of productive pre-mRNA over non-productive lncRNA.

We examined whether histone methyltransferase SETD2 is involved in lncRNA induction in SPT6-depeleted cells since SPT6 depletion redistributed H3K36me3 modification onto lncRNA regions. SETD2 knockout (KO) U2OS cells (Pfister et al., 2014) were SPT6 depleted, and SETD2, SPT6, and H3K36me3 protein levels were assessed by western blot (Figure 3G). Interestingly, qRT-PCR analysis shows that SETD2 KO does not affect the lncRNA induction caused by SPT6 depletion (Figure 3H). This suggests that SETD2 is not needed for lncRNA induction. A faint H3K36me3 level can still be detected in SETD2 KO cells, implying the existence of another methyltransferase for H3K36me3 in human cells.

Extended lncRNAs Induced by SPT6 Depletion Are Chromatin Restricted

We next tested whether SPT6-induced lncRNAs are released into the nucleoplasm or remain chromatin associated. The *YWHAZ* PROMPT significantly increases in the chromatin but not in the nucleoplasmic fractions, following SPT6 knockdown (Figure 4A). Meta-profiles of chromatin and nucleoplasm RNA-seq datasets show that lncRNAs induced by SPT6 depletion are retained in the chromatin fraction (Figure 4B). Remarkably, inactivation of the exosome shows the opposite effect. Thus, depletion of EXOSC3 (a core component of this complex, labeled EX3 in figure) causes no change to the levels of the *YWHAZ* PROMPT in chromatin but a marked accumulation in the nucleoplasm as confirmed by meta-analysis. We also performed a quantitative analysis of these effects, genome-wide by calculating the chromatin retention index for PROMPTs following either SPT6 or EXOSC3 depletion (Figure 4C). Notably, SPT6 depletion increases their chromatin retention index, while EXOSC3 depletion reduces this index.

PROMPTs are normally short transcripts in the range of 0.5–1 kb, and, in particular, exosome depletion causes their nucleoplasmic accumulation, as seen for the *YWHAZ* PROMPT (Figure 4A). Notably, SPT6 depletion not only causes chromatin-specific accumulation of this PROMPT, but also a substantial extension of this normally short transcript to a much longer >20 kb RNA. This is especially evident in the transcript profile seen with mNET-seq/T4P termination. For protein-coding genes, this profile is normally termination centric, but instead for *YWHAZ* PROMPT it is spread across the whole lncRNA TU (Schlackow et al., 2017) (Figure 4D). We also tested whether this highly extended and nuclear restricted lncRNA is polyadenylated. We show by both 3′ RNA-seq, which detects polyadenylated 3′ ends, and poly(A)+ RNA-seq that these RNAs are indeed polyadenylated (Figure 4D), presumably through recognition by CPA that is normally restricted to protein-coding gene transcripts. Quantitation of the effect of SPT6 depletion on lncRNA polyadenylation more generally shows a significant increase in polyadenylated 3′ ends for both PROMPTs and eRNA (Figures 4E and 4F). This double effect on both chromatin transcript accumulation and 3′ extension of lncRNA is particularly notable for the *MYC* locus (Figure S4A). While the short *MYC* TU are relatively unaffected by SPT6 or EXOC3 depletion in either chromatin or nuclear analyses, the antisense PROMPT is drastically altered both in size and amount. Thus, the *MYC* PROMPT was activated over 20-fold and extended for over 100 kb in the chromatin fraction. This suggests that SPT6 depletion induces lncRNA transcription and also disrupts the transcription termination. The extensive *MYC* PROMPT is also detectible using mNET-seq T4P and is polyadenylated with the activation of multiple PASs. This suggests that the ~150 kb *MYC* PROMPT is discontinuous. The same effect is detected in enhancers (Figure 4F), such as *NR4A1* eRNA regions (Figure S4B). SPT6 depletion induces and extends eRNA transcription on both sense and antisense strands. Finally, deregulated eRNAs are polyadenylated downstream of the RNA exosome-sensitive region, similarly to PROMPTs.

SPT6 Recruits the Integrator Complex to Terminate lncRNA Transcription

It is evident from our above results (Figures 3 and 4) that SPT6 depletion induces a general termination defect on lncRNA TUs.

(F) Boxplots of eRNA mNET-seq/total CTD and T4P signals at TSS (–/+2 kb).

(G and H) Western blot (G) and quantitative RT-PCR (H) of chromatin-bound RNA of parental and SETD2 CRISPR KO U2OS cells with indicated siRNA transfection for 48 hr. Data are represented as mean ± SEM.

See also Figure S3.

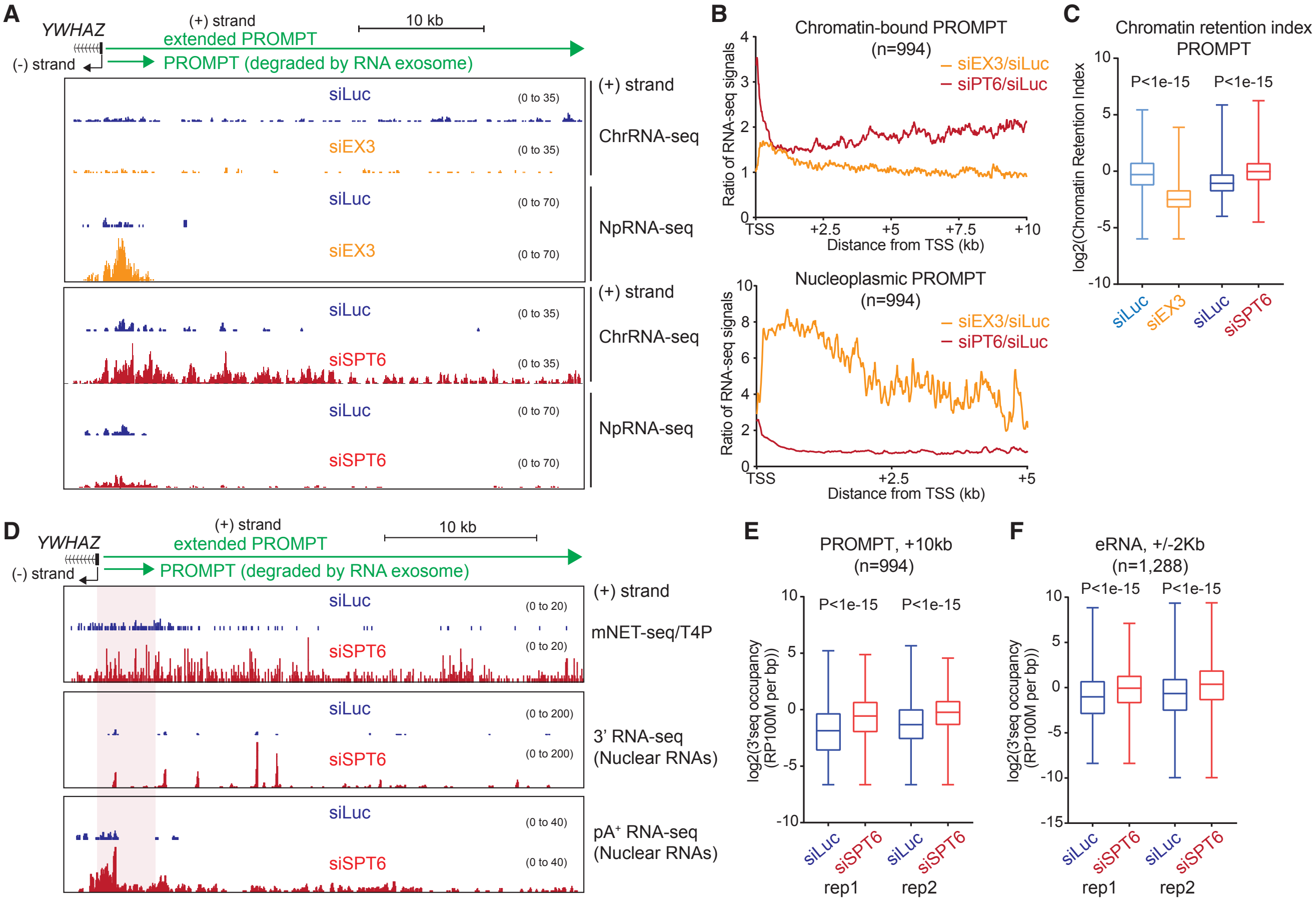

Figure 4. SPT6 Depletion Induces Extended, Chromatin-Restricted pA$^+$ lncRNAs

(A) *YWHAZ* PROMPT profile for strand-specific ChrRNA-seq and NpRNA-seq with EX3 or SPT6 siRNA-mediated depletions.
(B) Meta-analysis of ratio of siEX3 or siSPT6 over siLuc in chromatin (up to 10 kb from TSS) and nucleoplasm (up to 5 kb from TSS) RNA fractions.
(C) Chromatin retention indices of PROMPTs (up to 5 kb from TSS) following siLuc, siEX3, or siSPT6 depletions.
(D) *YWHAZ* PROMPT profile with mNET-seq/T4P, 3′ RNA-seq, and pA$^+$ nuclear RNA-seq.
(E) Boxplot replicates of 3′ RNA-seq at PROMPTs (10 kb from TSS) of divergent (PROMPT-pre-mRNA) genes.
(F) Boxplot of two replicates of 3′ RNA-seq signals at eRNA regions (2 kb from center).
See also Figure S4.

A known player in lncRNA termination is the large Integrator complex (Baillat and Wagner, 2015). This was first identified as a termination complex associated with U snRNA genes (Baillat et al., 2005) and has more recently also been associated with eRNA transcriptional termination (Lai et al., 2015).

We tested the possibility that SPT6 plays a role in Integrator recruitment to lncRNA genes by performing ChIP-seq using an antibody against the INTS3 component of the Integrator on HeLa cell chromatin with or without SPT6 depletion. As predicted, significant peaks of INTS3 ChIP-seq signal are detected over enhancer regions (generating bidirectional eRNA) that are substantially decreased following SPT6 depletion (Figure 5A). These data are resonant with a recent study showing that SPT6 is recruited to super enhancer regions in mouse embryonic stem cells (ESCs) (Wang et al., 2017). We also detect INTS3 ChIP-seq peaks over the TSS regions of divergent TUs (PROMPT-pre-mRNA), which are reduced by SPT6 depletion (Figure 5B). These results indicate that SPT6 is necessary to recruit this complex to lncRNA TSS. To further clarify these observations, we reanalyzed published Pol II ChIP-seq data obtained for INTS11-depleted HeLa cells (Figures 5C and 5D). As previously described, INTS11 depletion caused an increase in TSS-associated Pol II for both eRNA. We now describe the increase in PROMPT regions as well (Figure 5C). Notably, no effect of INTS11 depletion was observed across the body of protein-coding genes TUs even though their TSS peaks were affected. This is consistent with the proposed role of the Integrator complex in NELF-mediated Pol II pausing at the TSS of protein-coding genes (Stadelmayer et al., 2014). Quantitation of Pol II ChIP-seq by measurement of Pol II occupancy shows that INTS11 depletion significantly reduced the level of Pol II termination over PROMPT and eRNA TUs (Figure 5D). Overall, these molecular and bioinformatic analyses lead us to propose the following model for lncRNA termination (Figure 5E). In wild-type cells, SPT6 facilitates recruitment of The Integrator complex to promote cleavage of lncRNA near their TSS. This generates short pA$^-$ lncRNA. In contrast in SPT6-depleted cells, Pol II

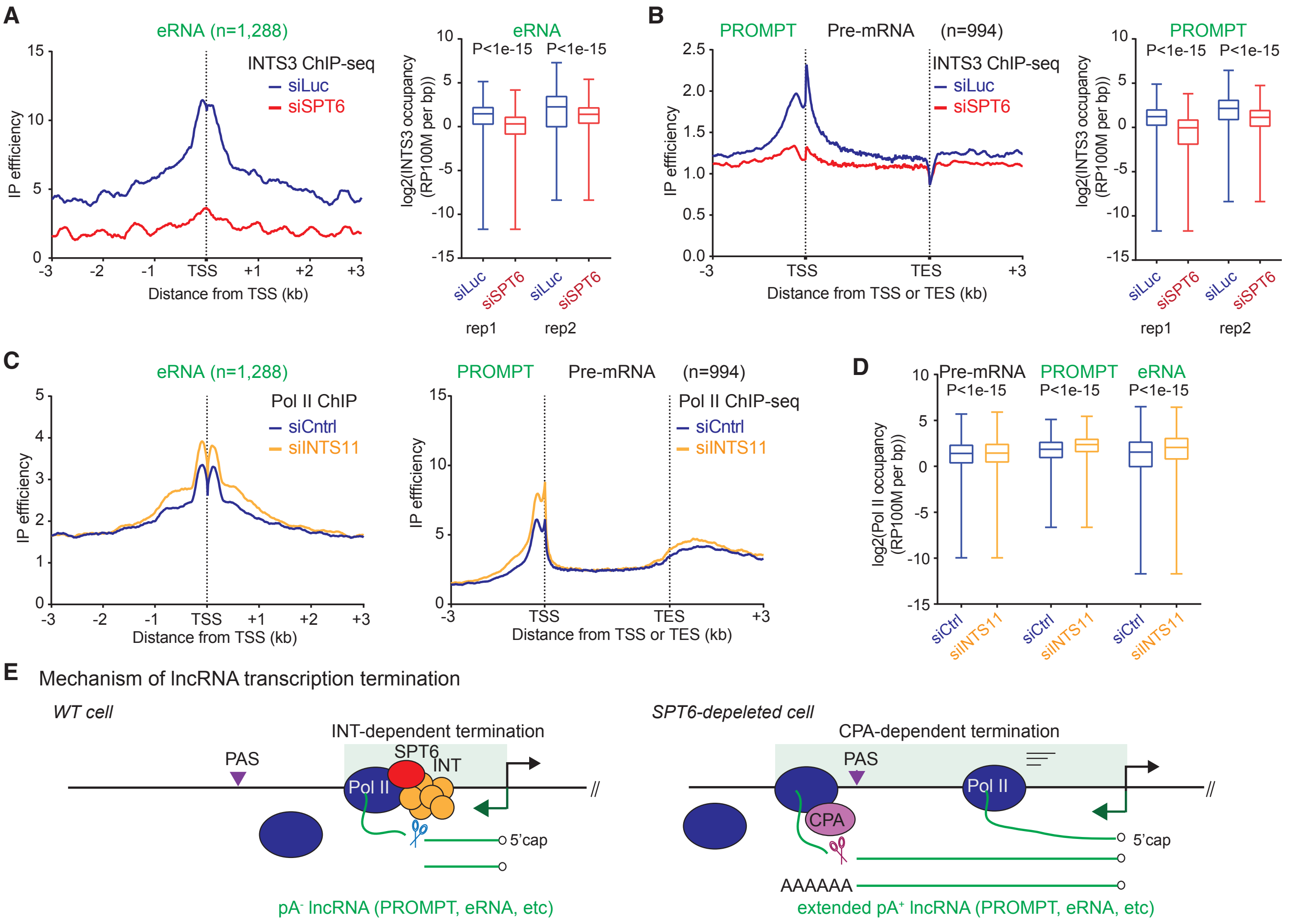

Figure 5. SPT6 Depletion Reduces INTS3 Recruitment

(A) Left: meta-analysis of INT3 ChIP-seq at eRNA regions (±3 kb from center). Right: boxplot replicates of INT3 ChIP-seq over eRNA.

(B) Left: meta-analysis of INTS3 ChIP-seq of divergent (PROMPT-pre-mRNA) genes. Right: boxplot replicates of INT3 ChIP-seq on PROMPTs.

(C) Left: meta-analysis of Pol II ChIP-seq over eRNA (left) and divergent (PROMPT-pre-mRNA) genes (right) upon INTS11 depletion. Reanalyzed data are from Stadelmayer et al. (2014).

(D) Boxplots of Pol II ChIP-seq signals upon INTS11 knockdown at pre-mRNA (whole annotated gene, n = 994), PROMPTs (3 kb from TSS, n = 994), and eRNA regions (2 kb from center, n = 1,288).

(E) Model of lncRNA transcription termination in WT and SPT6-depleted cells. SPT6 recruits Integrator (INT) complex to terminate lncRNA transcription so generating pA$^-$ lncRNA. Loss of SPT6 prevents INT recruitment, causing termination defects with extended pA$^+$ lncRNA, utilizing cleavage and polyadenylation (CPA) complex.

fails to recruit The Integrator complex, resulting in extended transcripts that are ultimately processed by CPA at cryptic PAS. This will result in the formation of polyadenylated transcripts coupled with downstream termination.

lncRNA Induction by SPT6 Depletion Induces R-Loop Accumulation

Our above results demonstrate that SPT6 depletion specifically induces lncRNA transcription that is both substantially length extended and chromatin restricted. A likely feature of such aberrant transcription is the induction of R-loop structures. These are RNA:DNA hybrids caused by the nascent transcript invading the DNA duplex behind elongating Pol II. This will result in displacement of the non-template DNA strand as single strand (ss) DNA. While R-loops play multiple roles in the modulation of gene expression (Santos-Pereira and Aguilera, 2015), they may also induce replication blocks due to the inability of the replication fork to read across regions of extended R-loop structure (Hamperl et al., 2017). In addition, the ssDNA of R-loops is inherently unstable. These combined features of R-loops can cause localized DNA damage (Skourti-Stathaki and Proudfoot, 2014). Although R-loops are potentially formed behind all elongating Pol II, transcript packaging or processing and especially the splicing complex may restrict their formation (Bonnet et al., 2017). In contrast, lncRNAs are generally inefficiently spliced (Schlackow et al., 2017) so that their transcripts may have a higher tendency to generate R-loops.

We performed genome-wide analyses of R-loops from HeLa cells with or without SPT6 depletion using a procedure involving native genomic DNA isolation and fragmentation by sonication.

Following immunoprecipitation with the RNA:DNA hybrid-specific antibody S9.6 (Boguslawski et al., 1986), hybrid RNA was cDNA amplified and sequenced to provide a strand-specific genome-wide R-loop profile (S.M.T.W., S.D., and N.J.P., unpublished data). We refer to this technology as RNA-DNA immunoprecipitation (DIP) sequencing (RDIP-seq). For *YWHAZ* and *DUSP1*, R-loop peaks were evident over their genic regions, as is generally observed for mammalian protein-coding genes (Ginno et al., 2013; Sanz et al., 2016), which were unaffected by SPT6 depletion (Figures 6A and S5A). In contrast their PROMPTs showed patches of R-loop signal over the enhanced and extended lncRNA TUs following SPT6 depletion, but very little signal prior to SPT6 depletion. Presumably, the discontinuous nature of these R-loop profiles reflects selective formation and stabilities of different R-loop regions. The upstream enhancer region of *DUSP1* also showed selective R-loop formation (Figure S5A). Remarkably only the eRNA transcribing toward *DUSP1* displayed R-loop signals (at least within a 4-kb window), and again these were greatly stimulated by SPT6 depletion. The absence of detectible R-loops in the antisense direction reflects an interesting specificity for this enhancer. In other cases, such as the *NR4A1* 90-kb downstream enhancer, increased R-loops signals were detected on both sense and antisense strands following SPT6 depletion (Figure 6B). Importantly, most RDIP-seq peaks were lost following RNase H treatment, and also the RNase H sensitivity on individual candidate gene loci was validated by DIP-qPCR analysis. This indicates that our RDIP-seq detects mainly R-loops, but not RNA species such as double-strand RNAs (Figures S5B and S5C).

To illustrate the generality of lncRNA R-loop formation, we show by Venn diagrams that the genome-wide signal distribution of RDIP-seq was increased over intergenic regions following SPT6 depletion, especially in upstream regions of protein-coding TUs (Figure 6C). This contrasts with the more abundant R-loop signal over genic (especially exon) sequences, which slightly decreased following SPT6 depletion. Overall, the induction of R-loops genome-wide following SPT6 depletion occurs selectively on PROMPT and eRNA regions, but not on protein-coding genes (Figures 6D).

SPT6 Depletion Induces DNA Damage and Cellular Senescence

Our final evaluation of the consequences of SPT6 depletion on the mammalian transcriptome led us to test for genetic and cellular defects as R-loops are well known to promote DNA damage. We therefore investigated the effect of SPT6 depletion on the levels of the DNA damage marker γ-H2AX (Turinetto and Giachino, 2015). Immunofluorescence analysis of HeLa cell nuclei showed a 6-fold accumulation of γ-H2AX foci indicative of DNA damage in SPT6-depleted cells (Figure 6E). To further extend these data, the distribution of γ-H2AX on chromatin following SPT6 depletion was established using ChIP-seq. Notably, higher signals were obtained over PROMPT and eRNA regions compared to protein-coding genes (Figure 6F). To focus on positions where R-loop peaks accumulate following SPT6 depletion, we compared the peak summits of RDIP-seq and γH2AX ChIP-seq signals on the lncRNA regions (Figure S5D) with individual examples of gene loci (Figure S5E) and showed that they substantially overlap. This correlation for RDIP and γH2AX signals holds true for both PROMPTs and eRNA. Note that randomly selected genomic positions that lack R-loops signals did not show γH2AX accumulation (Figure S5D). Additionally, γH2AX foci induced by SPT6 depletion were significantly reduced in number and intensity by transient overexpression of GFP-RNase H1 (Figure S5F). Taken together, our results demonstrate that SPT6 restricts lncRNA transcription from the human genome and thereby prevents R-loop formation and consequent DNA damage.

We observed above (Figures 3 and S3) that, while lncRNA transcription is activated following SPT6 depletion, protein-coding gene transcription appears to be reduced due to elongation defects. However, we reasoned that while some protein-coding genes should display reduced expression levels, genes associated with the DNA damage response might become activated during the SPT6 depletion period. Such a response would be necessary to counteract R-loop-induced DNA damage. To measure the effect of SPT6 depletion on the distribution of steady-state mRNA, we performed differential expression sequencing (DESeq2: [Love et al., 2014]) of pA^+ nuclear RNA in SPT6-depeleted cells (Figure S6A). While the nuclear mRNA levels of most protein-coding genes were unchanged during the 60-hr SPT6 depletion experiment, 1,716 mRNAs were downregulated, including SPT6 (directly targeted by siRNA treatment). This may reflect a class of mRNA with more rapid degradation kinetics. In contrast, 2,722 mRNAs were significantly upregulated based on DESeq2 analysis (Table S1). In particular, the cyclin-CDK inhibitor genes *CDKN1A*, *CDKN1C*, and *CDKN2B* were upregulated at both transcriptional (Figures S6B) and protein levels (Figure 7A). Note that transcription of many other genes such as *BRCA1* was downregulated following SPT6 depletion (Figure S6B). P21 (*CDKN1A*) and P57 (*CDKN1C*) are important for the cell-cycle transition from G1-S and G2-M (Besson et al., 2008). Consistent with a cell-cycle defect, fluorescence-activated cell sorting (FACS) of HeLa cells depleted for SPT6 (as compared to mock-treated with siLuc) showed reduced cell numbers in S phase but more in G2. This indicates that SPT6-depleted cells display G1-S and G2-M transition defects (Figure S6C). In order to confirm that induction of lncRNA transcription is an upstream event to cell-cycle arrest, we performed a time-course experiment of SPT6 siRNA transfection, measuring the levels of P21 and P57 proteins and a candidate lncRNA at each time point by western blot and qRT-PCR, respectively (Figures S6D and S6E). Reduced levels of SPT6 and induction of *YWHAZ* PROMPT were observed by 12 hr. In contrast, the upregulation of P21 and P57 shows a slower kinetic response, being strongly activated only after 36 hr of SPT6 depletion. This result suggests that deregulated lncRNA transcription leads to cell-cycle arrest.

We noticed that SPT6-depleted cells display a larger and flatter morphology, consistent with a senescence phenotype (Figure 7B) (Muñoz-Espín and Serrano, 2014). Since senescent cells display increased lysosome size, detectible by increased β-galactosidase (β-gal) activity at pH 6.0 (Kurz et al., 2000), we measured this with or without SPT6 depletion. Notably, SPT6-depleted cells with β-gal staining were readily detectible by microscopy, and increased β-gal activity was confirmed by cell

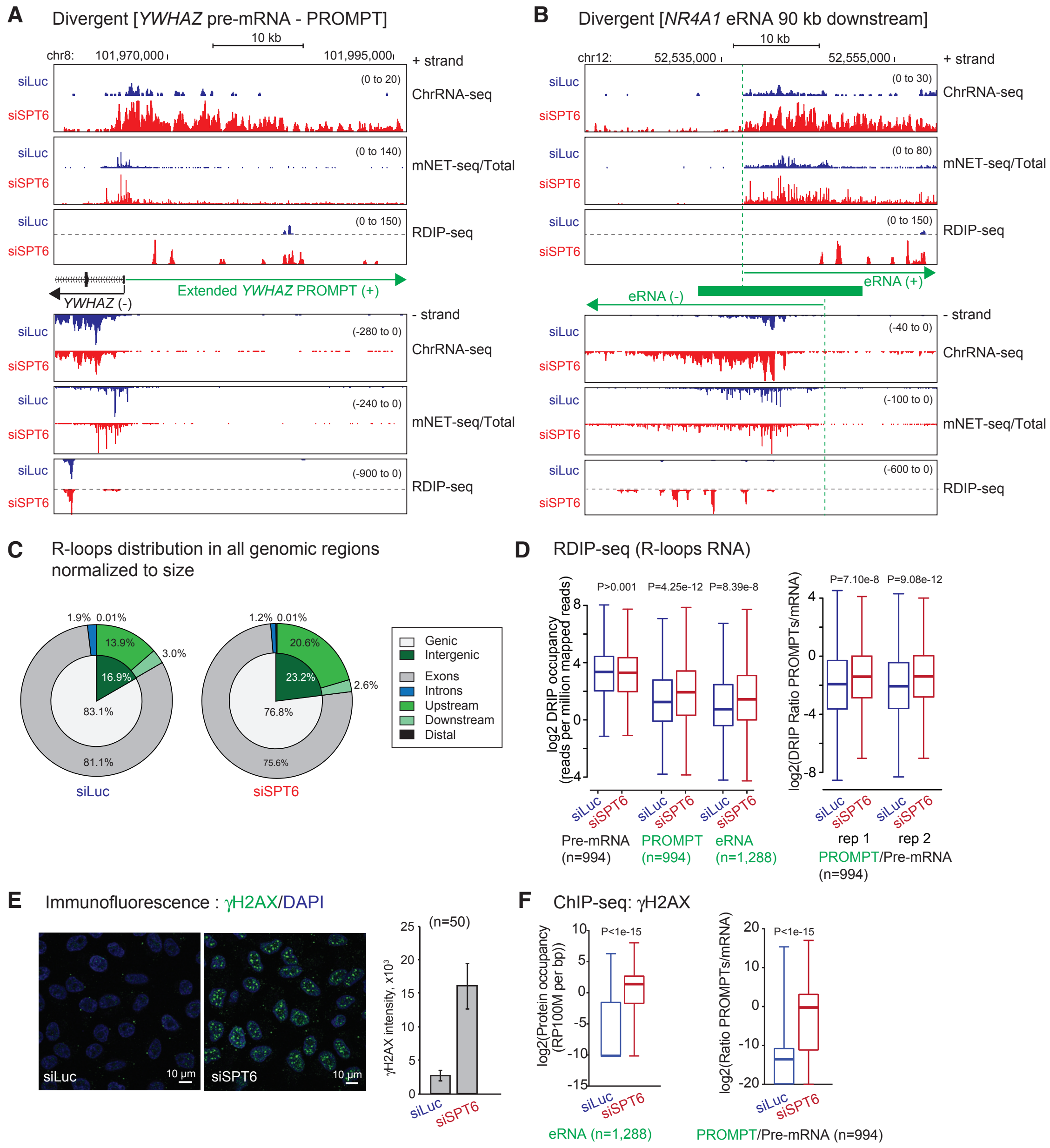

Figure 6. SPT6 Depletion Induces R-Loops and DNA Damage over lncRNA

(A) *YWHAZ* pre-mRNA gene (– strand, black arrow) and the PROMPT (+ strand, green arrow) showing RNA-DNA immunoprecipitation (RDIP)-seq profiles compared to ChrRNA-seq and mNET-seq/total CTD following SPT6 depletion.

(B) *NR4A1* eRNA RDIP-seq profiles compared to ChrRNA-seq and mNET-seq/total.

(C) Pie charts of RDIP-seq signal distribution for all genomic-associated regions (normalized to size) of control and SPT6-depleted HeLa cells. Genic versus Intergenic regions in inner layer. Exon and intron region of genic region and also upstream (< −2 kb from TSS), downstream (< +2 kb from TES), and distal (> −2 kb from TSS and > +2 kb from TES) of intergenic region shown in outer layer.

(D) Left: quantification of RDIP-seq signals over pre-mRNA (< +3 kb from TSS, n = 994), PROMPT (< −3 kb from TSS), and at eRNA (–/+ 3 kb from TSS) regions. Right: two replicates of RDIP-seq ratio of PROMPT and pre-mRNA regions.

(E) Left: immunofluorescence assay using anti γH2AX (green) in siLuc or siSPT6-transfected HeLa cells. DAPI (blue) for DNA staining. Right: γH2AX signal intensity from five fields of ten cells. Data are represented as mean ± SEM.

(F) Left: boxplots of γH2AX ChIP-seq signals at eRNA regions (2 kb from the center). Right: boxplot ratios of PROMPT over pre-mRNA signals from γH2AX ChIP-seq (3 kb from TSS).

See also Figure S5.

CellPress

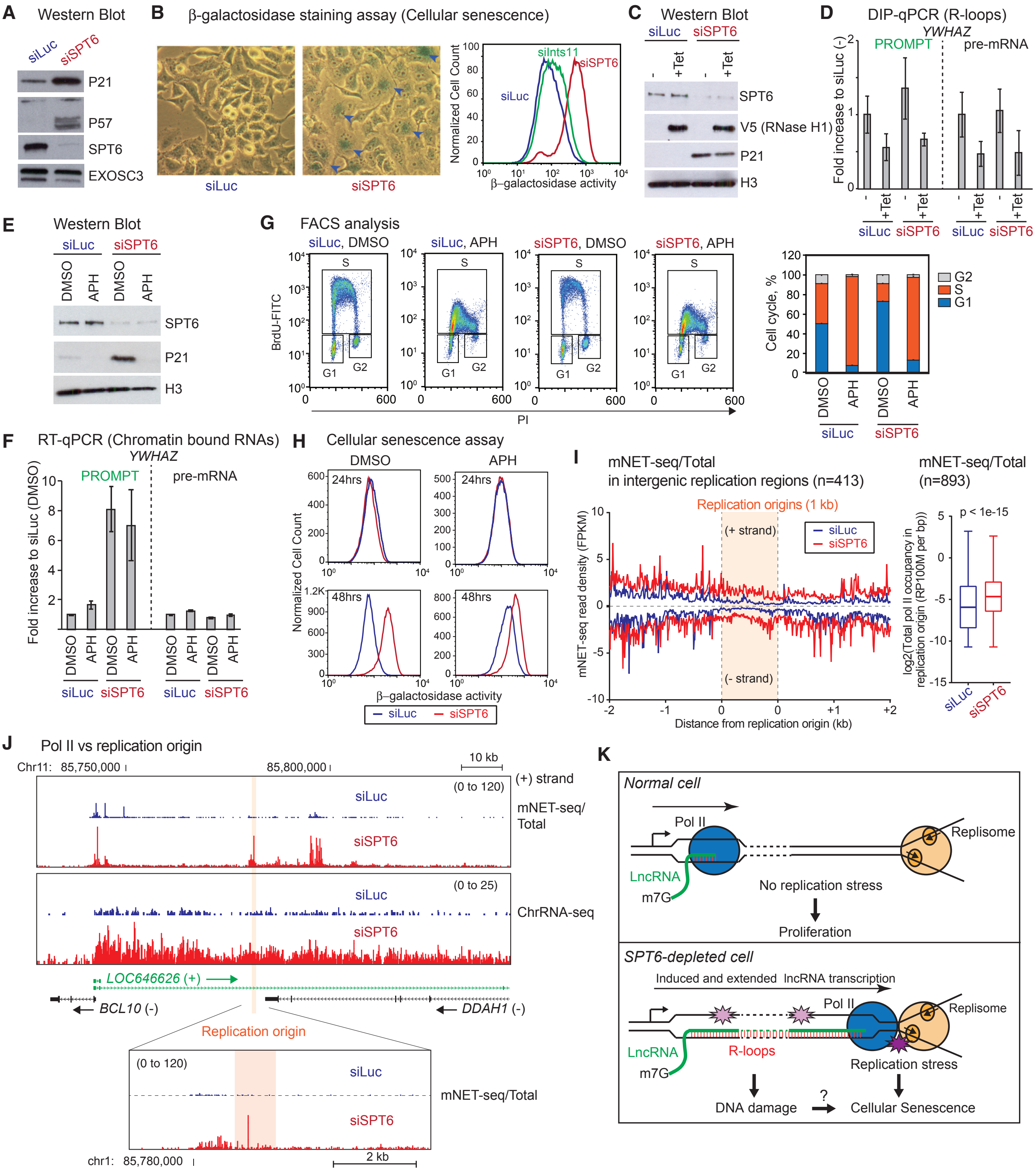

Figure 7. SPT6 Depletion Induces DNA Replication Stress and Cellular Senescence

(A) Western blot of whole-cell extract using indicated antibodies, following HeLa cell SPT6 depletion. EXOSC3 is loading control.

(B) Microscopy of β-galactosidase staining cells (blue) and associated quantification of β-galactosidase staining cells with SPT6 or INTS11 depletions. Blue arrowhead identifies β-gal positive cells.

(C) Western blot with indicated antibodies of engineered HeLa cells ± Tet-inducible V5-RNase H1 overexpression. After 4 hr siRNA transfection, fresh media ±1 μg/mL tetracycline (Tet) was added and incubated for 16 hr.

(D) DIP-qPCR analysis of *YWH2* pre-mRNA and PROMPT ± Tet induction of V5 RNase H1. Data are represented as mean ± SEM.

(legend continued on next page)

sorting. Quantification of this activity indicated that INTS11 depletion increased β-gal to a lesser extent than depletion of SPT6 (Figure 7B). This suggests that a solely termination defect for lncRNA transcription caused by loss of the Integrator complex is insufficient to arrest the cell cycle. In contrast, loss of SPT6, which both enhanced lncRNA transcription and causes loss of Integrator recruitment, does induce a significant cell-cycle arrest (Figure 5). We elected to investigate the correlation between R-loops and the cellular senescence phenotype in HeLa cells, since RNase H overexpression reduced the levels of DNA damage (Figure S5F). We therefore engineered a stable HeLa cell line overexpressing RNase H1. Induction of tagged V5-RNase H1 was detected by western blot (Figure 7C) correlating with an ~60% loss of R-loop signal from candidate lncRNA and pre-mRNA genes (Figure 7D). However, P21 levels were not significantly suppressed by V5-RNase H1 overexpression (Figure 7C). Although R-loops promote DNA damage induced by SPT6 depletion, a correlation between R-loops and cellular senescence remains unclear since remaining R-loops following RNase H1 overexpression may still be sufficient to induce P21 expression.

We describe in these studies the extensive induction of lncRNA transcription following SPT6 depletion. This results in increased levels of elongating Pol II in intergenic regions, a part of the genome that is normally transcriptionally silent. Such Pol II redistribution might lead to increased collision between Pol II and the DNA replisome that could cause of the observed DNA replication stress, DNA damage, and cellular senescence. We next examined the potential effect of DNA replication on the cellular senescence induced by SPT6 depletion by treating siRNA-transfected HeLa cells with a DNA polymerase inhibitor aphidicolin (APH). Notably, the senescence marker protein P21, activated by SPT6 depletion was suppressed by APH (Figure 7E) suggesting that activation of cellular senescence by SPT6 depletion is replication dependent. However, APH treatment did not affect chromatin RNA levels of *YWHAZ* pre-mRNA or PROMPT in SPT6-depleted cells (Figure 7F). The effect of APH treatment on DNA replication was confirmed by FACS, which showed a cell-cycle block in early S phase due to inhibition of nascent DNA synthesis in both control and SPT6-depleted cells (Figure 7G). Our results therefore indicate that DNA replication does indeed collide with deregulated Pol II in S phase. In addition, β-gal activity was induced by SPT6 siRNA only after 48 hr (Figure 7H). This argues that cellular senescence is a downstream event to the deregulation of lncRNA, P21, and the cell cycle. As induction of β-gal activity was significantly suppressed by APH (Figure 7H), this suggests that SPT6 depletion induces cellular senescence in a replication-dependent manner.

We finally compared the positions of DNA replication origins in HeLa cells with our mNET-seq data to look for a correlation between Pol II elongation and DNA replisome. Notably, most constitutive origins map to intergenic regions in the HeLa cell genome (Macheret and Halazonetis, 2018). While mNET-seq signals are very faint over intergenic DNA replication origin regions of control HeLa cells, these significantly increase following SPT6 depletion (Figure 7I; Table S2). In detail, 74 and 865 origins are located less that 100 kb away from a PROMPT or eRNA respectively and about half of these origins overlap with extended lncRNA induced by depletion of SPT6 (Figure S6F). It is evident that eRNA are particularly prone to collision with replication origins following SPT6 depletion. Examples of lincRNA, PROMPTs, and eRNA that overlap with replication origins following SPT6 depletion are presented (Figures 7J and S6G). Notably, mNET-seq signals induced by SPT6 depletion overlap with a replication origin in the promoter-associated lincRNA gene, *LOC646626* (Figure 7J), and suggest that Pol II pauses at this position due to collision with the DNA replisome. Overall, our results connect intergenic transcription-replication conflict with DNA stress and cellular senescence.

DISCUSSION

The widespread transcription of lncRNA across the human genome remains a surprising discovery of modern transcriptomic analysis. However, the expression of many lncRNAs (except for mRNA-like lincRNA) is restricted, implying that their accumulation may be deleterious to the cell. We now reveal a molecular explanation for this phenomenon by showing that SPT6 plays a pivotal role in the selectivity of protein-coding gene expression. Most protein-coding genes display a transcriptional elongation defect in SPT6-depleted cells as originally defined in *S. cerevisiae* (Hartzog et al., 1998). This is likely caused by defective recruitment of elongation factors. One such factor, ISW1 directly interacts with SPT6 and helps recruit SETD2 the methyltransferase required for H3K36me3 formation (Yoh et al., 2008). In contrast, in SPT6-depleted cells all major classes of lncRNA, PROMPT, eRNA, and lincRNA (except for the mRNA-like category), display enhanced nascent transcription levels and generate extended transcripts due to loss of the Integrator

(E) Western blot with indicated antibodies of whole-cell extract from HeLa cells ± SPT6, +/− following 18 hr aphidicolin (APH) treatment.

(F) qRT-PCR of *YWHAZ* chromatin-bound RNA from cells as in (E). Data are represented as mean ± SEM.

(G) FACS of HeLa cells (+/− SPT6 depletion). After 6 hr with siRNA DMSO (control) or APH was added for 18 hr. Quantitation shown on the right is mean ± SEM.

(H) Quantification of β-galactosidase-stained cells treated with DMSO or APH. Top: siRNA transfection (6 hr) and then DMSO or APH added and incubated (18 hr). Total was 24 hr. Bottom: siRNA transfection (24 hr) and then DMSO or APH added and incubated (24 hr). Total was 48 hr.

(I) Left: meta-analysis of mNET-seq/total CTD in –/+ 2-kb regions from HeLa DNA replication origins (1 kb). Right: boxplots quantifying mNET-seq/total CTD signals over HeLa DNA replication origins.

(J) LincRNA LOC646626 gene loci (+ strand) as example of mNET-seq and ChrRNA-seq profiles over DNA replication origin. Magnified field of replication origin shown below.

(K) Model: collision between Pol II and DNA replisome in SPT6-depleted cell. Pol II and DNA replisome are separated in wild-type cells. In SPT6-depleted cells, induced and extended lncRNA transcription in intergenic region causes deregulated Pol II collision with DNA replisome. R-loops may also cause DNA damage and cellular senescence.

See also Figure S6 and Tables S1 and S2.

complex recruitment. It still remains unclear how the Integrator complex is recruited by SPT6.

Previous analysis of SPT6 revealed that it is an essential and ubiquitous protein conserved from yeast (Bortvin and Winston, 1996) to mammals. Interestingly, in human cell lines, UV irradiation causes a Pol II elongation defect (Williamson et al., 2017) reminiscent of SPT6 depletion. Indeed, UV treatment promotes degradation of SPT6 via the ubiquitin-proteasome pathway (Boeing et al., 2016). This suggests that SPT6 is likely to be at least partially involved in the well-established UV-mediated Pol II elongation defect. Additionally, knockdown of the proteasome modulator PAAF1 induced SPT6 protein degradation though ubiquitination, resulting in loss of Tat-mediated HIV-1 transcription (Nakamura et al., 2012). Exactly how SPT6 regulates Pol II transcription activity on lncRNA genes remains unclear. In yeast, SPT6 is thought to cooperate with the DRB-sensitivity-inducing factor (DSIF) complex (SPT4 and SPT5) since mutations in all three genes show similar phenotypes (Hartzog et al., 1998; Swanson and Winston, 1992; Winston et al., 1984). Notably, SPT5 depletion in *S. pombe* has been observed to restrict promoter-proximal transcription in protein-coding genes and at the same time enhances antisense transcription initiating on either side of Pol II promoters (Shetty et al., 2017). In contrast, in mammalian cells, SPT5 depletion appears to restrict promoter proximal transcription equally for both protein-coding and lncRNA genes (Henriques et al., 2018). This may suggest differences in the transcriptional role of SPT5 between yeast and mammals.

The fact that lncRNAs are not in general subjected to co-transcriptional RNA processing (splicing and polyadenylation) led us to test whether they promote DNA damage through R-loop formation following their activation by SPT6 depletion. This appeared especially likely as it has been previously observed that loss of the splicing factor SRSF1 correlates with increased R-loop formation in chicken DT40 cells (Li and Manley, 2005). Also, recent studies confirm that disease-related mutations of other splicing factors such as SRSF2 and U2AF1 enhance R-loop formation in mammals (Chen et al., 2018). Furthermore, the spliceosome was observed to prevent R-loops formation on intron-containing pre-mRNA genes especially in *S. cerevisiae* (Bonnet et al., 2017). Similarly, the low levels of intron splicing for lncRNA (Schlackow et al., 2017) implies an increased risk of R-loop formation. Our analysis of R-loop profiles across the HeLa cell genome does indeed show a selective increase in their presence over lncRNA TUs. Furthermore, this increase correlates with increased levels of DNA damage. However, R-loop-specific RNase H1 overexpression did not suppress the cellular senescence even though DNA damage was reduced as predicted from earlier studies (Skourti-Stathaki and Proudfoot, 2014). The biological importance of R-loops for senescence remains unclear. However, overexpression of RNase H1 does not fully remove R-loops *in vivo*, possibly because associated factors may restrict access to the enzyme. Indeed, recent R-loop proteomic analyses show that numerous proteins bind to R-loop regions (Cristini et al., 2018).

Interestingly, inhibition of DNA replication by APH significantly suppressed the cellular senescence phenotype. This led us to consider the possibility that SPT6 depletion causes the extensive collision of lncRNA transcription with DNA replication (Figure 7K). Normally, Pol II transcription of lncRNA, such as eRNA, is restricted to near their TSSs (Lai and Shiekhattar, 2014). However, about 80% of high confidence replication origins identified in a recent analysis (Macheret and Halazonetis, 2018) map to intergenic regions within 100 kb of lncRNA promoters (Figures 7I and S6F) and nearly half of these origins overlap with extended lncRNA (especially eRNA) formed by SPT6 depletion. In effect, SPT6 acts to prevent such collision and so maintains genome stability. These observations also underline the requirement that lncRNA TUs and especially eRNA must be properly restricted to allow normal cell growth.

Exactly how loss of SPT6 enhances H3K36me3 disposition on lncRNA genes is also unresolved. It is conceivable that the histone methyltransferase complex PRC2 known to catalyze the gene silencing mark H3K27me3 (Margueron and Reinberg, 2011) also regulates H3K36me3 levels on lncRNA genes. Possibly SPT6 recruits distinct complexes to protein-coding (SETD2) and lncRNA genes (PRC2). This may imply that Pol II can distinguish coding or noncoding TUs by their histone methylation status. H3K27me3 marks and SPT6 appear to be mutually exclusive in mouse ESCs (Wang et al., 2017). Similarly, our mNuc-seq data show that genome-wide profiles of H3K27me3 and H3K36me3 are strongly anti-correlated in HeLa cells (Figure S1C). These observations suggest that SPT6 plays an important role in maintaining H3K36me3 on highly expressed protein-coding genes. Overall, it is clear that SPT6 acts as a master regulator of Pol II elongation, favoring productive protein coding over non-productive and potentially deleterious lncRNA transcription.

STAR★METHODS

Detailed methods are provided in the online version of this paper and include the following:

- KEY RESOURCES TABLE
- PRIMERS FOR (RT-)QPCR
- CONTACT FOR REAGENT AND RESOURCE SHARING
- EXPERIMENTAL MODEL AND SUBJECT DETAILS
- METHOD DETAILS
  - siRNA transfection
  - APH treatment
  - mNET-seq method and library prep
  - Chromatin-bound RNA (ChrRNA) and Nucleoplasm RNA (NpRNA)-seq methods and library preparation
  - Mononucleosome DNA (mNuc)-seq method and the library prep
  - Chromatin immunoprecipitation sequencing (ChIP-seq) and library preparation
  - pA^+ RNA-seq and 3′RNA-seq methods and the library preps
  - DIP-qPCR and RDIP-seq
  - Fluorescence activated cell sorting (FACS)
  - Quantitative β-galactosidase assay
  - Immunofluorescent staining assay
  - Normalized RT-qPCR with *Drosophila* cells
  - Establishment of V5-RNase H1 overexpressing HeLa cell line

- QUANTIFICATION AND STATISTICAL ANALYSIS
  - mNET-seq and RNA-seq data processing
  - mNuc-seq and ChIP-seq data processing
  - 3′RNA-seq data processing
  - RDIP-seq data processing
  - RDIP signal distribution
  - mNuc-seq correlation heatmap
  - TU annotation
  - Metagene profiles
  - Differential expression analysis
  - Reads quantification
  - Chromatin retention index
  - Location of DNA replication origin
  - Spatial analysis of DNA replication origins
  - P values and significance tests
- DATA AND SOFTWARE AVAILABILITY

SUPPLEMENTAL INFORMATION

Supplemental Information includes six figures and two tables and can be found with this article online at https://doi.org/10.1016/j.molcel.2018.10.011.

ACKNOWLEDGMENTS

We thank members of the N.J.P. and S.M. groups for critical discussion. We also thank Dr. Fumiko Esashi for helpful advice on assays for DNA damage and cellular senescence. This work was supported by funding to N.J.P. (Wellcome Trust Investigator Award [107928/Z/15/Z] and ERC Advanced [339270] grants) and to S.M. (Wellcome Trust Investigator Award [WT106134AIA]).

AUTHOR CONTRIBUTIONS

T.N. performed all molecular biology experiments with J.F. for mNuc-seq, C.R.d.A. for FACS, A.D. for immunofluorescence analysis, and S.M.T.W. for RDIP-seq. G.D. generated the HeLa cell expressing Tet-ON pro-V5-RNase H1. M.T. carried out all bioinformatic analyses with S.D. for RDIP-seq. T.N., S.M., and N.J.P. designed the project and wrote the paper.

DECLARATION OF INTERESTS

The authors declare no competing interests.

Received: February 16, 2018
Revised: July 13, 2018
Accepted: October 9, 2018
Published: November 15, 2018

REFERENCES

Almada, A.E., Wu, X., Kriz, A.J., Burge, C.B., and Sharp, P.A. (2013). Promoter directionality is controlled by U1 snRNP and polyadenylation signals. Nature *499*, 360–363.

Andersson, R., Gebhard, C., Miguel-Escalada, I., Hoof, I., Bornholdt, J., Boyd, M., Chen, Y., Zhao, X., Schmidl, C., Suzuki, T., et al. (2014). An atlas of active enhancers across human cell types and tissues. Nature *507*, 455–461.

Baillat, D., and Wagner, E.J. (2015). Integrator: Surprisingly diverse functions in gene expression. Trends Biochem. Sci. *40*, 257–264.

Baillat, D., Hakimi, M.A., Näär, A.M., Shilatifard, A., Cooch, N., and Shiekhattar, R. (2005). Integrator, a multiprotein mediator of small nuclear RNA processing, associates with the C-terminal repeat of RNA polymerase II. Cell *123*, 265–276.

Bannister, A.J., and Kouzarides, T. (2011). Regulation of chromatin by histone modifications. Cell Res. *21*, 381–395.

Besson, A., Dowdy, S.F., and Roberts, J.M. (2008). CDK inhibitors: Cell cycle regulators and beyond. Dev. Cell *14*, 159–169.

Boeing, S., Williamson, L., Encheva, V., Gori, I., Saunders, R.E., Instrell, R., Aygün, O., Rodriguez-Martinez, M., Weems, J.C., Kelly, G.P., et al. (2016). Multiomic analysis of the UV-induced DNA damage response. Cell Rep. *15*, 1597–1610.

Boguslawski, S.J., Smith, D.E., Michalak, M.A., Mickelson, K.E., Yehle, C.O., Patterson, W.L., and Carrico, R.J. (1986). Characterization of monoclonal antibody to DNA.RNA and its application to immunodetection of hybrids. J. Immunol. Methods *89*, 123–130.

Bonnet, A., Grosso, A.R., Elkaoutari, A., Coleno, E., Presle, A., Sridhara, S.C., Janbon, G., Geli, V., de Almeida, S.F., and Palancade, B. (2017). Introns protect eukaryotic genomes from transcription-associated genetic instability. Mol. Cell *67*, 608–621.

Bortvin, A., and Winston, F. (1996). Evidence that Spt6p controls chromatin structure by a direct interaction with histones. Science *272*, 1473–1476.

Calo, E., and Wysocka, J. (2013). Modification of enhancer chromatin: What, how, and why? Mol. Cell *49*, 825–837.

Cerritelli, S.M., Frolova, E.G., Feng, C., Grinberg, A., Love, P.E., and Crouch, R.J. (2003). Failure to produce mitochondrial DNA results in embryonic lethality in Rnaseh1 null mice. Mol. Cell *11*, 807–815.

Chen, Y., Pai, A.A., Herudek, J., Lubas, M., Meola, N., Järvelin, A.I., Andersson, R., Pelechano, V., Steinmetz, L.M., Jensen, T.H., and Sandelin, A. (2016). Principles for RNA metabolism and alternative transcription initiation within closely spaced promoters. Nat. Genet. *48*, 984–994.

Chen, L., Chen, J.Y., Huang, Y.J., Gu, Y., Qiu, J., Qian, H., Shao, C., Zhang, X., Hu, J., Li, H., et al. (2018). The augmented R-loop is a unifying mechanism for myelodysplastic syndromes induced by high-risk splicing factor mutations. Mol. Cell *69*, 412–425.

Chiu, A.C., Suzuki, H.I., Wu, X., Mahat, D.B., Kriz, A.J., and Sharp, P.A. (2018). Transcriptional pause sites delineate stable nucleosome-associated premature polyadenylation suppressed by U1 snRNP. Mol. Cell *69*, 648–663.e7.

Cristini, A., Groh, M., Kristiansen, M.S., and Gromak, N. (2018). RNA/DNA hybrid interactome identifies DXH9 as a molecular player in transcriptional termination and R-loop-associated DNA damage. Cell Rep. *23*, 1891–1905.

DeGennaro, C.M., Alver, B.H., Marguerat, S., Stepanova, E., Davis, C.P., Bähler, J., Park, P.J., and Winston, F. (2013). Spt6 regulates intragenic and antisense transcription, nucleosome positioning, and histone modifications genome-wide in fission yeast. Mol. Cell. Biol. *33*, 4779–4792.

Endoh, M., Zhu, W., Hasegawa, J., Watanabe, H., Kim, D.K., Aida, M., Inukai, N., Narita, T., Yamada, T., Furuya, A., et al. (2004). Human Spt6 stimulates transcription elongation by RNA polymerase II in vitro. Mol. Cell. Biol. *24*, 3324–3336.

Ginno, P.A., Lim, Y.W., Lott, P.L., Korf, I., and Chédin, F. (2013). GC skew at the 5′ and 3′ ends of human genes links R-loop formation to epigenetic regulation and transcription termination. Genome Res. *23*, 1590–1600.

Hamperl, S., Bocek, M.J., Saldivar, J.C., Swigut, T., and Cimprich, K.A. (2017). Transcription-replication conflict orientation modulates R-loop levels and activates distinct DNA damage responses. Cell *170*, 774–786.

Hartzog, G.A., Wada, T., Handa, H., and Winston, F. (1998). Evidence that Spt4, Spt5, and Spt6 control transcription elongation by RNA polymerase II in Saccharomyces cerevisiae. Genes Dev. *12*, 357–369.

Henriques, T., Scruggs, B.S., Inouye, M.O., Muse, G.W., Williams, L.H., Burkholder, A.B., Lavender, C.A., Fargo, D.C., and Adelman, K. (2018). Widespread transcriptional pausing and elongation control at enhancers. Genes Dev. *32*, 26–41.

Kaida, D., Berg, M.G., Younis, I., Kasim, M., Singh, L.N., Wan, L., and Dreyfuss, G. (2010). U1 snRNP protects pre-mRNAs from premature cleavage and polyadenylation. Nature *468*, 664–668.

Kurz, D.J., Decary, S., Hong, Y., and Erusalimsky, J.D. (2000). Senescence-associated (beta)-galactosidase reflects an increase in lysosomal mass during replicative ageing of human endothelial cells. J. Cell Sci. *113*, 3613–3622.

Lai, F., and Shiekhattar, R. (2014). Enhancer RNAs: The new molecules of transcription. Curr. Opin. Genet. Dev. *25*, 38–42.

Lai, F., Gardini, A., Zhang, A., and Shiekhattar, R. (2015). Integrator mediates the biogenesis of enhancer RNAs. Nature *525*, 399–403.

Li, X., and Manley, J.L. (2005). Inactivation of the SR protein splicing factor ASF/SF2 results in genomic instability. Cell *122*, 365–378.

Li, W., Notani, D., and Rosenfeld, M.G. (2016). Enhancers as non-coding RNA transcription units: Recent insights and future perspectives. Nat. Rev. Genet. *17*, 207–223.

Love, M.I., Huber, W., and Anders, S. (2014). Moderated estimation of fold change and dispersion for RNA-seq data with DESeq2. Genome Biol. *15*, 550.

Macheret, M., and Halazonetis, T.D. (2018). Intragenic origins due to short G1 phases underlie oncogene-induced DNA replication stress. Nature *555*, 112–116.

Margueron, R., and Reinberg, D. (2011). The Polycomb complex PRC2 and its mark in life. Nature *469*, 343–349.

Marquardt, S., Raitskin, O., Wu, Z., Liu, F., Sun, Q., and Dean, C. (2014). Functional consequences of splicing of the antisense transcript COOLAIR on FLC transcription. Mol. Cell *54*, 156–165.

Mieczkowski, J., Cook, A., Bowman, S.K., Mueller, B., Alver, B.H., Kundu, S., Deaton, A.M., Urban, J.A., Larschan, E., Park, P.J., et al. (2016). MNase titration reveals differences between nucleosome occupancy and chromatin accessibility. Nat. Commun. *7*, 11485.

Mukherjee, N., Calviello, L., Hirsekorn, A., de Pretis, S., Pelizzola, M., and Ohler, U. (2017). Integrative classification of human coding and noncoding genes through RNA metabolism profiles. Nat. Struct. Mol. Biol. *24*, 86–96.

Muñoz-Espín, D., and Serrano, M. (2014). Cellular senescence: From physiology to pathology. Nat. Rev. Mol. Cell Biol. *15*, 482–496.

Nakamura, M., Basavarajaiah, P., Rousset, E., Beraud, C., Latreille, D., Henaoui, I.S., Lassot, I., Mari, B., and Kiernan, R. (2012). Spt6 levels are modulated by PAAF1 and proteasome to regulate the HIV-1 LTR. Retrovirology *9*, 13.

Nojima, T., Gomes, T., Grosso, A.R.F., Kimura, H., Dye, M.J., Dhir, S., Carmo-Fonseca, M., and Proudfoot, N.J. (2015). Mammalian NET-seq reveals genome-wide nascent transcription coupled to RNA processing. Cell *161*, 526–540.

Nojima, T., Gomes, T., Carmo-Fonseca, M., and Proudfoot, N.J. (2016). Mammalian NET-seq analysis defines nascent RNA profiles and associated RNA processing genome-wide. Nat. Protoc. *11*, 413–428.

Nojima, T., Rebelo, K., Gomes, T., Grosso, A.R., Proudfoot, N.J., and Carmo-Fonseca, M. (2018). RNA polymerase II phosphorylated on CTD serine 5 interacts with the spliceosome during co-transcriptional splicing. Mol. Cell *72*, 369–379.

Ntini, E., Järvelin, A.I., Bornholdt, J., Chen, Y., Boyd, M., Jørgensen, M., Andersson, R., Hoof, I., Schein, A., Andersen, P.R., et al. (2013). Polyadenylation site-induced decay of upstream transcripts enforces promoter directionality. Nat. Struct. Mol. Biol. *20*, 923–928.

Pfister, S.X., Ahrabi, S., Zalmas, L.P., Sarkar, S., Aymard, F., Bachrati, C.Z., Helleday, T., Legube, G., La Thangue, N.B., Porter, A.C., and Humphrey, T.C. (2014). SETD2-dependent histone H3K36 trimethylation is required for homologous recombination repair and genome stability. Cell Rep. *7*, 2006–2018.

Sandelin, A., Carninci, P., Lenhard, B., Ponjavic, J., Hayashizaki, Y., and Hume, D.A. (2007). Mammalian RNA polymerase II core promoters: Insights from genome-wide studies. Nat. Rev. Genet. *8*, 424–436.

Santos-Pereira, J.M., and Aguilera, A. (2015). R loops: New modulators of genome dynamics and function. Nat. Rev. Genet. *16*, 583–597.

Sanz, L.A., Hartono, S.R., Lim, Y.W., Steyaert, S., Rajpurkar, A., Ginno, P.A., Xu, X., and Chédin, F. (2016). Prevalent, dynamic, and conserved R-loop structures associate with specific epigenomic signatures in mammals. Mol. Cell *63*, 167–178.

Schlackow, M., Nojima, T., Gomes, T., Dhir, A., Carmo-Fonseca, M., and Proudfoot, N.J. (2017). Distinctive patterns of transcription and RNA processing for human lincRNAs. Mol. Cell *65*, 25–38.

Sdano, M.A., Fulcher, J.M., Palani, S., Chandrasekharan, M.B., Parnell, T.J., Whitby, F.G., Formosa, T., and Hill, C.P. (2017). A novel SH2 recognition mechanism recruits Spt6 to the doubly phosphorylated RNA polymerase II linker at sites of transcription. eLife *6*. Published online August 16, 2017. https://doi.org/10.7554/eLife.28723.

Shetty, A., Kallgren, S.P., Demel, C., Maier, K.C., Spatt, D., Alver, B.H., Cramer, P., Park, P.J., and Winston, F. (2017). Spt5 plays vital roles in the control of sense and antisense transcription elongation. Mol. Cell *66*, 77–88.

Skourti-Stathaki, K., and Proudfoot, N.J. (2014). A double-edged sword: R loops as threats to genome integrity and powerful regulators of gene expression. Genes Dev. *28*, 1384–1396.

Skourti-Stathaki, K., Proudfoot, N.J., and Gromak, N. (2011). Human senataxin resolves RNA/DNA hybrids formed at transcriptional pause sites to promote Xrn2-dependent termination. Mol. Cell *42*, 794–805.

St Laurent, G., Wahlestedt, C., and Kapranov, P. (2015). The landscape of long noncoding RNA classification. Trends Genet. *31*, 239–251.

Stadelmayer, B., Micas, G., Gamot, A., Martin, P., Malirat, N., Koval, S., Raffel, R., Sobhian, B., Severac, D., Rialle, S., et al. (2014). Integrator complex regulates NELF-mediated RNA polymerase II pause/release and processivity at coding genes. Nat. Commun. *5*, 5531.

Swanson, M.S., and Winston, F. (1992). SPT4, SPT5 and SPT6 interactions: Effects on transcription and viability in Saccharomyces cerevisiae. Genetics *132*, 325–336.

Turinetto, V., and Giachino, C. (2015). Multiple facets of histone variant H2AX: A DNA double-strand-break marker with several biological functions. Nucleic Acids Res. *43*, 2489–2498.

Ulitsky, I., and Bartel, D.P. (2013). lincRNAs: Genomics, evolution, and mechanisms. Cell *154*, 26–46.

Voong, L.N., Xi, L., Sebeson, A.C., Xiong, B., Wang, J.P., and Wang, X. (2016). Insights into nucleosome organization in mouse embryonic stem cells through chemical mapping. Cell *167*, 1555–1570.

Vos, S.M., Farnung, L., Boehning, M., Wigge, C., Linden, A., Urlaub, H., and Cramer, P. (2018). Structure of activated transcription complex Pol II-DSIF-PAF-SPT6. Nature *560*, 607–612.

Wang, A.H., Juan, A.H., Ko, K.D., Tsai, P.F., Zare, H., Dell'Orso, S., and Sartorelli, V. (2017). The elongation factor Spt6 maintains ESC pluripotency by controlling super-enhancers and counteracting polycomb proteins. Mol. Cell *68*, 398–413.

Whitehouse, I., Rando, O.J., Delrow, J., and Tsukiyama, T. (2007). Chromatin remodelling at promoters suppresses antisense transcription. Nature *450*, 1031–1035.

Williamson, L., Saponaro, M., Boeing, S., East, P., Mitter, R., Kantidakis, T., Kelly, G.P., Lobley, A., Walker, J., Spencer-Dene, B., et al. (2017). UV irradiation induces a non-coding RNA that functionally opposes the protein encoded by the same gene. Cell *168*, 843–855.

Winston, F., Chaleff, D.T., Valent, B., and Fink, G.R. (1984). Mutations affecting Ty-mediated expression of the HIS4 gene of Saccharomyces cerevisiae. Genetics *107*, 179–197.

Yoh, S.M., Lucas, J.S., and Jones, K.A. (2008). The Iws1:Spt6:CTD complex controls cotranscriptional mRNA biosynthesis and HYPB/Setd2-mediated histone H3K36 methylation. Genes Dev. *22*, 3422–3434.

STAR★METHODS

KEY RESOURCES TABLE

REAGENT OR RESOURCE	SOURCE	IDENTIFIER
Reagent		
APH	Santa Cruz	Cat# sc-201535
Tetracycline (Tet)	Sigma	Cat# 87128
Primers		
Random primers	Thermo Fisher	Cat# 48190011
Drosophila positive control primer set	Active motif	Cat# 71037
siRNAs		
siLuc (custom siRNA)	Sigma	Sequence (5′-3′) Sense: GAUUAUGUCCGGUUAUGUAUU Antisense: [phos]UACAUAACCGGACAUAAUCUU
siSPT6 (human), SMART pool, ON-TARGETplus	Dharmacon (GE)	L-010540-00-0010
Antibodies		
Mouse monoclonal anti-Pol II CTD, Total	MBL international	Cat# MABI0601; RRID: AB_2728735
Mouse monoclonal anti-Pol II CTD, phospho Ser2	MBL international	Cat# MABI0602; RRID: AB_2747403
Mouse monoclonal anti-Pol II CTD, phospho Ser5	MBL international	Cat# MABI0603; RRID: AB_2728736
Rat monoclonal anti-Pol II CTD, phospho Thr4 (6D7)	Active Motif	Cat# 61361; RRID: AB_2750848
Mouse monoclonal anti-Trimethyl Histone H3 (Lys36)	MBL international	Cat# MABI0333; RRID: AB_11126731
Mouse monoclonal anti-Trimethyl Histone H3 (Lys27)	MBL international	Cat# MABI0323; RRID: AB_11123929
Mouse monoclonal anti-Trimethyl Histone H3 (Lys4)	MBL international	Cat# MABI0304; RRID: AB_11123891
Mouse monoclonal anti-Monomethyl Histone H3 (Lys4)	MBL international	Cat# MABI0302; RRID: AB_11126551
Mouse monoclonal anti-H3	MBL international	Cat# MABI0301; RRID: AB_11142498
Rabbit polyclonal anti-Spt6	Novus Biologicals	Cat# NB100-2582; RRID: AB_2196402
Rabbit polyclonal anti-EXOSC3	Novus Biologicals	Cat# NBP2-22261; RRID: AB_2750849
Rabbit polyclonal anti- INTS3	Proteintech	Cat# 16620-1-AP; RRID: AB_2127274
Rabbit monoclonal anti-p57 Kip2 [EP2515Y]	Abcam	Cat# ab75974; RRID: AB_1310535
Rabbit monoclonal anti-p21 Waf1/Cip1 (12D1)	Cell Signaling	Cat# 2947; RRID: AB_823586
Mouse monoclonal anti-phospho Histone H2A.X Ser139, (JBW301)	Millipore	Cat# 05-636; RRID: AB_309864
Mouse monoclonal anti-RNA:DNA hybrids, (S9.6)	Proudfoot Lab	N/A; RRID: AB_2750851
Mouse monoclonal anti-V5 antibody	Thermo Fisher	Cat# R960-25; RRID: AB_2556564
Rabbit polyclonal anti-SETD2 antibody	Abcam	Cat#ab69836; RRID: AB_2185782
Deposited Data		
Raw sequencing data	This paper	GEO: GSE110028
Re-analyzed ChIP-seq data	(Stadelmayer et al., 2014)	GEO: GSE60586
Re-analyzed Chromatin and Nucleosplasmic RNA-seq	(Schlackow et al., 2017)	GEO: GSE81662
RDIP-seq data	This paper	GEO: GSE120371
Re-analyzed EdU-seq data	(Macheret and Halazonetis, 2018)	SRA: PRJNA397123
Raw image data	Mendeley	https://doi.org/10.17632/gtgh75y4ct.1
Cell Lines		
HeLa (human)	Proudfoot Lab	N/A
U2OS (human)	(Pfister et al., 2014)	Parental and SETD2 KO cells are available from Humphrey Lab by request.
V5-RNase H1 overexpressing HeLa (human)	this study	N/A

(*Continued on next page*)

Continued		
REAGENT OR RESOURCE	SOURCE	IDENTIFIER
Gels		
Novex 6% TBE gel, 12 well	Invitrogen	Cat# EC62652BOX
Novex 6% TBE-Urea (TBU) gel, 12 well	Invitrogen	Cat# EC68652BOX
Kits		
Dynabeads mRNA Purification kit	Ambion	Cat# 61006
Superscript III first strand synthesis system	Thermo Fisher	Cat# 18080051
SensiMix SYBR Non ROX kit	Bioline	Cat# QT650
Ribo-Zero Gold rRNA removal kit (H/M/R)	Illumina	Cat# MRZG12324
Senescence β-gal staining kit	Cell Signaling	Cat# 9860
Quantitative cellular senescence assay kit (SA- β-gal, Fluorometric)	Cell Biolabs	Cat# CBA-232
NEBNext Ultra II DNA library prep kit for illumina	NEB	Cat# E7645S
NEBNext Ultra II Directional RNA library prep kit for illumina	NEB	Cat# E7760S
NEBNext small RNA library prep kit for Illumina	NEB	Cat# E7300S
QuantSeq 3′mRNA-Seq library prep kit REV for Illumina	LEXOGEN	Cat# SKU016.24.
Software and Algorithms		
Cutadapt(v1.9.1)		https://cutadapt.readthedocs.io/en/stable/installation.html
Tophat(v2.1.0)		http://ccb.jhu.edu/software/tophat/index.shtml
Cufflinks(v2.2.0)		http://cole-trapnell-lab.github.io/cufflinks/getting_started/
bedtools (v2.25.0)		https://bedtools.readthedocs.io/en/latest/content/installation.html
Bowtie2 (v2.2.5)		http://bowtie-bio.sourceforge.net/bowtie2/index.shtml
SAMtools (v1.6)		http://www.htslib.org/
Picard (v1.131)		http://broadinstitute.github.io/picard/
Deeptools (v2.5.3)		https://deeptools.readthedocs.io/en/latest/index.html
HTseq (v0.6.1)		https://htseq.readthedocs.io/en/release_0.9.1/#

PRIMERS FOR (RT-)qPCR

PRIMER’S NAME	PRIMER’S SEQUENCES (5′-3′)
YWHAZ PROMPT_FW	GAGTGCTGGCTAATGGGGTA
YWHAZ PROMPT_RV	CTGGGAATCCTCTCCATTCA
YWHAZ_pre-mRNA_FW	CCCATCAAGTTGCCTCCATA
YWHAZ_pre-mRNA_RV	CCAAGGACAATCACGACCTT
CCND1 PROMPT_FW	AGCAGCCCTTCTCCCTAGAC
CCND1 PROMPT_RV	GGATAAAGGGCCTCTCCTTG
CCND1_pre-mRNA_FW	TGAAGAATCCCTGGATGGAG
CCND1_pre-mRNA_RV	GCCTGGGGTGAGATACAAGA
NR4A1 eRNA_FW	CAGCAATGGGGCCTTGTAGA
NR4A1 eRNA_RV	CAAGTTCCAACGGGCAACAG

CONTACT FOR REAGENT AND RESOURCE SHARING

Further information and requests for resources and reagents should be directed to the lead contact, Nicholas Proudfoot (nicholas.proudfoot@path.ox.ac.uk).

EXPERIMENTAL MODEL AND SUBJECT DETAILS

HeLa and U2OS cells were maintained in high glucose Dulbecco's Modified Eagle's Medium (DMEM) with 10% fetal bovine serum (FBS).

METHOD DETAILS

siRNA transfection

siRNAs against luciferase and human SPT6 (final concentration 30 nM) were transfected into HeLa cells using Lipofectamine RNAiMAX reagent (Life technologies) according to the manual and incubated for 12-60 hr.

APH treatment

APH powder (sc-201535) was dissolved in DMSO as stock solution 2mM. After 6 hr siRNA transfection in HeLa cells, DMEM was replaced with APH (final 2 μM) or DMSO (control, 0.1%) and incubated for 18 hr. The APH treated cells are analyzed by western blot, qRT-PCR and FACS.

mNET-seq method and library prep

mNET-seq was carried out as previously described (Nojima et al., 2016) with minor changes. In brief, the chromatin fraction was isolated from $1x10^7$ HeLa cells. Chromatin was digested in 100 μL of MNase (40 units/ μL) reaction buffer for 3-5 min at 37°C in a thermomixer (1,400 rpm). After addition of 10 μL EGTA (25mM) to inactivate MNase, soluble digested chromatin was collected by 13,000 rpm centrifuge for 5 min. The supernatant was diluted with 400 μL of NET-2 buffer and Pol II antibody-conjugated beads were added. 10 μg of Pol II antibody was used for Total and T4P CTD mNET-seq experiments. Immunoprecipitation was performed at 4°C for 1 hr. The beads were washed with 1 mL of NET-2 buffer six times with 100 μL of 1xPNKT (1xPNK buffer and 0.05% Triton X-100) buffer once in cold room. Washed beads were incubated in 50 μL PNK reaction mix (1xPNKT, 1 mM ATP and 0.05 U/ml T4 PNK 3′phosphatase minus (NEB) in Thermomixer (1,400 rpm) at 37°C for 6 min. After the reaction beads were washed with 1 mL of NET-2 buffer once and RNA was extracted with Trizol reagent. RNA was suspended in urea Dye (7M Urea, 1xTBE, 0.1% BPB and 0.1% XC) and resolved on 6% TBU gel (Invitrogen) at 200 V for 5 min. In order to size select 30-160 nt RNAs, a gel fragment was cut between BPB and XC dye markers. 0.5 mL tube was prepared with 3-4 small holes made with 25G needle and placed in a 1.5 mL tube. Gel fragments were placed in the layered tube and broken down by centrifugation at 12,000 rpm for 1 min. The small RNAs were eluted from gel using RNA elution buffer (1 M NaOAc and 1 mM EDTA) at 25°C for 1 hr in Thermomixer (900 rpm). Eluted RNA was purified with SpinX column (Coster) with 2 glass filters (Millipore) and the flow-through RNA was ethanol precipitated. RNA libraries were prepared according to manual of NEBNext small RNA library prep kit (NEB). 12∼14 cycles of PCR were used to amplify the library. Deep sequencing (Hiseq4000, Illumina) was conducted by the high throughput genomics team of the Wellcome Trust Centre for Human Genetics (WTCHG), Oxford.

Chromatin-bound RNA (ChrRNA) and Nucleoplasm RNA (NpRNA)-seq methods and library preparation

Detailed protocols of ChrRNA and NpRNA-seqs are as previously described (Nojima et al., 2015). In brief, chromatin RNA fraction was prepared from SPT6-depleted HeLa cells (approximately $5x10^6$ cells) according protocol. Prior to RNA library preparations, rRNA was depleted using Ribo-Zero Glod rRNA removal kit (Illumina) from 5 μg of chromatin and nucleoplasmic RNA. Using 100 ng of RNA, libraries were made according to the NEBNext Ultra II Directional RNA Library Prep kit for Illumina (NEB) manual. 12∼14 cycles of PCR were used to amplify the library. Deep sequencing (Hiseq4000, Illumina) was conducted as above.

Mononucleosome DNA (mNuc)-seq method and the library prep

Chromatin fraction was isolated from $8x10^6$ HeLa cells according to ChrRNA-seq protocol (Nojima et al., 2015). The chromatin was digested in 100 μL of MNase (40 units/ μL) reaction buffer for 3-5 min at 37°C in thermomixer (1,400 rpm). After adding 10 μL of EGTA (25mM) to inactivate MNase, soluble digested chromatin was collected by 13,000 rpm centrifuge for 5 min. Solublized DNA fragments were purified using phenol/chloroform (pH 7.0) and ethanol precipitation. For input, the DNAs were incubated with RNase A (0.01 mg/mL, Ambion) at 37°C for 10 min and purified again using phenol/chloroform (pH 7.0) and ethanol precipitation. For histone marks, the solubilized chromatin fraction was IPed with 10 μg of various histone antibodies at 4°C for 1 hr. The IPed DNAs were washed with 1 mL NET-2 buffer six times. DNAs were purified as an input preparation and then size selected for 100-200 nt on 6% TBE gel (Invitrogen). Using 100 ∼500 ng of DNA, libraries were made according to the NEBNext Ultra II DNA Library Prep Kit for Illumina (NEB) manual. 5∼9 cycles of PCR were used to amplify the library. Deep sequencing (Hiseq4000, Illumina) was conducted as above

Chromatin immunoprecipitation sequencing (ChIP-seq) and library preparation

After ∼60 hr siRNA transfection in $8x10^6$ HeLa cells (10 mL DMEM), 260 uL of 36.5% formaldehyde was added to medium (10 ml) at 37°C for 10 min with gentle shaking. For inactivation, 1 mL of 1.32 M Glycine was added at 37°C for 10 min. Cells were washed with cold PBS twice and centrifuged at 1,400 rpm for 5 min to collect cells into 10 mL tube (Nunc). Washed cells were lysed with 300 μL of

Cell Lysis buffer (10 mM Tris-HCl pH8.0, 85 mM KCl, 0.5% NP-40 and 1xComplete) and incubated on ice for 10 min. They were then centrifuged at 2,400 rpm for 5 min to remove supernatant (cytoplasm fraction). Nuclear pellets were resuspended in 400 μL of Nuclear Lysis buffer (25 mM Tris-HCl pH8.0, 0.5% SDS, 5 mM EDTA and 1xComplete) and incubated on ice for 10 min. Next cell suspensions were sonicated for 20 min (medium power, 30 s on-off repeats). To collect 400 μL of supernatant as a soluble chromatin fraction, sonicated nuclei were centrifuged at 13,000 rpm for 10 min. Nucleosomes were then diluted 10 times with IP dilution buffer (10 mM Tris-HCl pH8.0, 5 mM EDTA, 0.5% Triton X-100 and 0.15 M NaCl) and isolated from supernatant by IP with 10 μg of various antibodies. IPed DNA was washed with 1 mL of buffer A (20 mM Tris-HCl pH 8.0, 2 mM EDTA, 0.05% SDS, 1% Triton X-100 and 0.165 M NaCl) once, 1 mL of buffer B (20 mM Tris-HCl pH8.0, 2 mM EDTA, 0.05% SDS, 1% Triton X-100 and 0.5 M NaCl) once, 1 mL of buffer C (10 mM Tris-HCl pH8.0, 1 mM EDTA, 1% NP-40, 1% Sodium Deoxycholate and 0.25 M LiCl) and then 1 mL of buffer D (10 mM Tris-HCl pH8.0 and 1 mM EDTA) twice. Next IPed beads were incubated with 0.01 mg/mL RNase A (Ambion) in 300 μL of buffer E (1% SDS, 0.1 M $NaHCO_3$ and 0.5 M NaCl) at 65°C for at least 4 hr. After RNase treatment, 30 μL of 10x Proteinase K mixture (200 mM Tris-HCl pH 6.5, 150 mM EDTA and Proteinase K 0.3 mg/mL) were added and then incubated 45°C for 2 hr. The DNA fragments were purified using phenol/chloroform (pH 7.0) and ethanol precipitation. DNA libraries were made according to NEBNext Ultara II DNA Library Prep Kit for Illumina (NEB) manual. 13∼15 cycles of PCR were used to amplify the library. Deep sequencing (Hiseq4000, Illumina) was conducted as above.

pA^+ RNA-seq and 3′RNA-seq methods and the library preps

pA^+ RNA was isolated from nuclear RNA fraction of SPT6-depeleted HeLa cells as previously described (Schlackow et al., 2017). For pA^+ RNA-seq, libraries were prepared from the pA^+ RNA fraction according to NEBNext Ultra II Directional RNA Library Prep kit for Illumina (NEB) manual. For 3′RNA-seq, libraries were prepared from the pA^+ RNA fraction according to QuantSeq 3′mRNA-Seq library prep kit REV for Illumina (LEXOGEN) manual. 50∼100 ng of pA^+ RNAs were used for the library preps. 13∼15 cycles of PCR were used to amplify the library. Hiseq4000 and Hiseq2500 (rapid mode) were used for deep sequencing of pA^+ RNA-seq and 3′RNA-seq libraries, respectively.

DIP-qPCR and RDIP-seq

RDIP-seq experiments were modified from previous method (Skourti-Stathaki et al., 2011). Briefly, nuclei isolated from HeLa cells from an 80% confluent 10 cm^2 plate were subjected to nuclear lysis after which nuclear extracts were incubated with 30 μg of proteinase K (Roche) at 55°C for 3 hr, and genomic DNA was isolated and quantitated. Genomic DNA was pre-treated with RNase I (Promega) at 10 U per 3ug genomic DNA for 15 min at 37°C before sonication (Bioruptor) to 200-300bp. Fragmented DNA was incubated overnight with S9.6 antibody. RNA-DNA hybrids were enriched by immuno-magnetic precipitation with M-280 sheep anti-mouse IgG Dynabeads (Invitrogen). They were then extracted by phenol/chloroform (Sigma) and precipitated in the presence of glycogen before resuspension in nuclease free water. Here, samples can be removed for DIP-qPCR analysis where specific primers were tested. For RDIP-seq, samples were then incubated at 90°C for 3 min and quick cooled to 4°C before subjecting to DNase I treatment. RNA moiety of the R-loop was then extracted with TRIreagent and precipitated with isopropanol and glycogen. Libraries were prepared with the NEBNext Ultra II Directional RNA Library Prep Kit for Illumina (NEB) according to the manufacturer's guidelines. Libraries were sequenced on an Illumina NEXTseq 550 with 75 bp single end reads.

Fluorescence activated cell sorting (FACS)

Cell cycle profiles were obtained by adding 10 μM of 5-bromo-2′-deoxyuridine (BrdU, Sigma-Aldrich) to HeLa cells 1 hr prior to harvesting. Single-cell suspensions were washed with PBS, fixed in ice-cold 70% ethanol and kept overnight at 4°C. For BrdU staining, cells were incubated in 2N HCl, 0.5% Triton X-100 for 30 min at room temperature followed by a 2 min incubation in 0.1 M sodium tetraborate buffer, pH 8.0. Cells were then washed with PBS, 1% BSA and incubated with FITC-conjugated anti-BrdU antibody (BioLegend) in PBS, 1% BSA, 0.5% Tween-20 for 1 hr at room temperature. They were then washed again in PBS and stained with 0.02 mg/mL propidium iodide (PI) in PBS, 0.1% Triton X-100, 0.2 mg/mL RNase A for 30 min at room temperature. Samples were acquired on a FACSCalibur flow cytometer (BD Biosciences) and analyzed with FlowJo software (Tree Star).

Quantitative β-galactosidase assay

For β-galatosidase activity measurement, single-cell suspensions of SPT6-depleted HeLa cells from 35 mm dish were stained with SA-β-GAL substrate according to manufacturer's instructions (Cell Biolab).

Immunofluorescent staining assay

In brief, HeLa cells were grown on a coverslip in a 6 well plate 24 hr prior to treatment. HeLa cells were treated with indicated siRNAs for 48 hr. For GFP-RNase H1 overexpression, plasmid transfection were performed at 24 hr post siRNA treatment, followed by incubation for a further 24 hr. HeLa cells were fixed with 4% PFA in PBS. Primary antibody anti-γH2A.X (JBW-301) was used at 1:200 in 3% BSA in PBS for 1 hr at room temperature. Cells were washed thrice with 0.05% Tween20-PBS followed by incubation with secondary donkey anti-mouse IgG (H+L) conjugated with Alexa Fluor 488 at (1:250) concentration. Z stack images were collected with a FluoView1000 confocal microscope (Olympus) using a UPLSAPO 60.0X / 1.35 oil objective. Images were analyzed using ImageJ and prepared using OMERO software. For γH2A.X foci quantification, approximately 10 unique fields of view from

distinct images, were captured at random. Binary images were thresholded and water shed once with area of each foci was determined using the ‘Analyze Particles’ feature of ImageJ. Cut-off of particle size was set to (> 200 nm2-infinity) and circularity (0.5-1.10). The percentage of foci > 200nm2 were scored as positive.

Normalized RT-qPCR with *Drosophila* cells

In order to normalized RT-PCR signals within different conditions, PBS-washed 0.1 million *Drosophila* S2 cells were added to PBS-washed 10 million HeLa or U2OS cells. From these mixed cells, chromatin-bound RNAs were purified (see above). 500 ng chromatin-bound RNAs were used with superscript III kit and random primers for cDNA synthesis according to the manufactural protocol. The cDNAs were amplified with indicated primers and *Drosophila* specific positive primer set for spike-in normalization. SensiMix kit was used for quantitative real-time PCR (QIAGEN Rotor-gene).

Establishment of V5-RNase H1 overexpressing HeLa cell line

pcDNA5-V5-RNaseH1 was constructed by sub-cloning the RNase H1 ORF from GFP-RNaseH1 vector (Cerritelli et al., 2003) into a pcDNA5 vector carrying a V5 tag. The pcDNA5-V5-RNaseH1 vector was co-transfected with the Flp-recombinase expression vector pOG44 into T-Rex HeLa cells. After 150 mg/mL Hygromycin B selection, a clone expressing high level of V5-RNase H1 with 1mg/mL tetracycline was isolated.

QUANTIFICATION AND STATISTICAL ANALYSIS

mNET-seq and RNA-seq data processing

mNET-seq and RNA-seq (chromatin, nucleoplasmic, cytoplasmic, and nuclear pA^{+}) data were processed as follows: adapters were trimmed with Cutadapt in paired-end mode with the following parameters: -q 15, 10 **–**minimum-length 10 -A GATCGTCGGACTGTAGAACTCTGAAC -a TGGAATTCTCGGGTGCCAAGG. Trimmed reads were mapped to the human hg19 reference sequence with Tophat2 and the parameters –g 1 –r 3000 –no-coverage-search. SAMtools was used to retain only properly paired and mapped reads (-f 3). For mNET-seq, a custom python script (Nojima et al., 2015) was used to obtain the 3′ nucleotide of the second read and the strandedness of the first read. Strand-specific bam files were generated with SAMtools. Library-size normalized bedgraph files were created with Bedtools (genomecov –bg –scale) and trackhubs in the UCSC browser were generated with the UCSC bedGraphToBigWig tool.

mNuc-seq and ChIP-seq data processing

Adapters were trimmed with Cutadapt in paired-end mode with the same parameters as mNET-seq. Obtained sequences were mapped to the human hg19 reference genome with Bowtie2. Properly paired and mapped reads were filtered with SAMtools. PCR duplicates were removed with Picard MarkDuplicates tool. Library-size normalized bedgraph files were created with Bedtools and trackhubs in the UCSC browser were generated with the UCSC bedGraphToBigWig tool.

3′RNA-seq data processing

Adapters were trimmed with BBduk in paired-end mode with the following parameters: k = 13 ktrim = r useshortkmers = t mink = 5 qtrim = t trimq = 10 minlength = 20 forcetrimleft = 11. Trimmed reads were mapped to the human hg19 reference sequence with Bowtie2. SAMtools was used to retain only properly paired and mapped reads (-f 3) and for creating strand-specific bam files. Library-size normalized bedgraph files were created with Bedtools and trackhubs in the UCSC browser were generated with the UCSC bedGraphToBigWig tool.

RDIP-seq data processing

Raw reads from RNA-DIP (RDIP)-seq were demultiplexed using in-house Perl script. Reads were aligned to reference genome hg19/GRCh37 using bowtie2.2.5. Uniquely mapped reads with one mismatch in the seed region (-N1 -k1) was allowed. Plus and minus strand were assigned to mapped reads using SAMtools. RDIP-seq peaks were called using MACS2 algorithm with default options. For siSPT6, peaks were called by using siLuc as control. Peaks with q-value below 0.05 were retained for further analysis. Strands were assigned to peaks by intersecting the called peaks to strand specific reads using bedtools.

RDIP signal distribution

Genome wide distribution of RDIP peaks was performed by calculating the expression of RDIP peaks relative to various RefSeq functional categories: exons, introns, upstream, downstream and distal. Upstream and downstream peaks were those that overlapped with −2kb of TSS and +2kb of TES respectively. Peaks overlapping with regions beyond these were considered as distal.

mNuc-seq correlation heatmap

The mNuc-seq heatmap was computed with Deeptools2 multiBamSummary tool with the following parameters: bins –bs 10000 –distanceBetweenBins 0 –p max –e. The resulting matrix was plotted with Deeptool2 plotCorrelation with the following parameters:–corMethod spearman –skipZeros –colorMap RdYlBu –plotNumbers.

TU annotation

Gencode V19 annotation, based on the hg19 version of the human genome, was used to extract TUs. All genes were taken from the most 5′ TSS to the most 3′ PAS or transcription end site (TES). The set of non-overlapping protein-coding genes was defined as followed: non-overlapping annotated feature upstream of downstream of the TSS or PAS, respectively, in a window of 2.5 kb. The TU must also be longer than 2 kb. Chromatin RNA-seq coverage for this group of genes was then clustered into four groups, based on the k-mean method, and the three most expressed groups were merged together to create final set of 2,500 non-overlapping protein coding genes. Intronless protein-coding genes were extracted from the Gencode V19 annotation by keeping the protein-coding genes containing a single exon and removing the histone genes because of their difference in transcriptional regulation. This results in a set of 1319 genes. snRNA genes were extracted from the Gencode V19 annotation by keeping all the genes and pseudogenes annotated as snRNA, resulting in 542 genes. PROMPTs, eRNAs, and mRNAs-mRNAs annotation in HeLa cells were previously classified (Chen et al., 2016). PROMPT-associated genes were obtained by extracting the nearest protein-coding gene on the opposite strand of each PROMPT, resulting in 994 mRNA-PROMPT pairs. mRNA-lincRNA pairs were extracted from the Gencode V19 data by keeping only the mRNA ("protein-coding" type) and lincRNAs ("lincRNA" and "antisense" types) on opposite strands and with their TSSs separated by less than 3 kb. Out of the 716 mRNA-lincRNA pairs, 594 were kept with at least 10 total Pol II mNET-seq reads in siLuc or siSPT6 conditions for both mRNA and lincRNAs.

Metagene profiles

FPKM normalized bigwig files were generated for each bam files with Deeptools2 bamCoverage tool (-bs 1 (mNET-seq) or 10 –p max –normalizeUsingRPKM –e (for mNuc-seq and ChIP-seq)). Metagene profiles were then generated with Deeptools2 computeMatrix tool with a bin size of 10 bp and the plotting data obtained with plotProfile –outFileNameData tool. Graphs were then created with GraphPad Prism 7.02.

Differential expression analysis

For differential expression analysis of nucleoplasmic and nuclear poly(A)$^+$ RNA-seq, the aligned reads were aggregated with htseq-count and the list of differentially expressed genes obtained with DESeq2, keeping only the genes with a fold change < -2 or > 2 and an adjusted p value of 0.05. RNA-seq smear plots showing average gene expression (x axis) versus log2 fold change in gene expression were produced with DEseq2.

Reads quantification

Total read base count for mNET-seq, RNA-seq and 3′seq data were computed with samtools bedcov tool using strand-specific bam files and normalized to 100 million paired-end reads and to the region's length. For mNuc-seq and ChIP-seq, total read base count were computed with samtools bedcov, normalized to 100 million paired-end reads, then the Input signal was subtracted to the IP signal and normalized to the region's length. Only the regions with a positive signal in at least one sample were kept. For the samples having a signal ≤ 0 on the remaining regions, their values were put to the minimal value divided by two. The quantification is thus defined: For mNET-seq RNA-seq, and 3′seq: log2(([Region] * normalization factor) / $length_{region}$). For mNuc-seq and ChIP-seq: log2((($[region]_{IP}$ * IP normalization factor) - ($[region]_{Input}$ * Input normalization factor)) / $length_{region}$) The quantification regions were defined for the different group of genes, except when indicated in the figures: for PROMPT, TSS to TSS + 3kb; eRNA, TSS -2 kb to TSS + 2kb; lincRNA, TSS to TES; protein-coding genes (jntron-containing and intronless): TSS to TES; snRNA: TSS to TES. Scatterplots, which represents the reads quantification in siLuc on the x axis and in siSPT6 on the y axis, and the box and whiskers, which were plotted with the minimal and maximal values, were created with GraphPad Prism 7.02.

Chromatin retention index

The Chromatin Retention Index (CRI) was computed from chromatin and nucleoplasmic RNA-seq after siSPT6 or siEX3 treatments. The total read base count across each PROMPT region, defined as TSS to TSS + 5 kb, was computed with samtools bedcov for the chromatin and nucleoplasmic RNA-seq. After normalization to 100 million paired-end reads and the region length, the chromatin signal was divided by the nucleoplasmic signal for each PROMPT region. The CRI was then defined as CRI = log2(($[TSS, TSS + 5\ kb]_{chromatin\ counts}$ * normalization factor) / 5000) / (($[TSS, TSS + 5\ kb]_{nucleoplasmic\ counts}$ * normalization factor) / 5000).

Location of DNA replication origin

The 1 kb locations of the annotated replication origin in HeLa cells were taken from previously published paper (Macheret and Halazonetis, 2018). Out of the 1,336 constitutive replication origins, only the 917 intergenic replication origins were kept. Quantification of the mNET-seq/Total CTD signal in siLuc and siSPT6 across the 1 kb window provided a list of 893 constitutive and intergenic replication origins with a positive signal for Pol II in at least one sample (Table S2). Among these 893 replication origins, 413 have Pol II signal increased by at least a 2-fold after SPT6 depletion (Table S2).

Spatial analysis of DNA replication origins

To determine for each intergenic replication origin the closest ncRNA, the location of the 917 intergenic replication origins were compared to the location of the 994 PROMPTs and of the 32,692 eRNAs annotated in the PrESSTo database, which is part of the FANTOM5 project.

P values and significance tests

P values were computed by a Wilcoxon rank sum test. Paired Wilcoxon signed rank test was compared. Statistical tests were performed in GraphPad Prism 7.02.

DATA AND SOFTWARE AVAILABILITY

The accession number for the RDIP-seq reported in this paper is GEO: GSE120371. The accession number for all other sequencing data reported in this paper is GEO: GSE60586. Original images of western blot, gel and immunofluorescent staining assay are available at Mendeley data https://doi.org/10.17632/gtgh75y4ct.1.

Molecular Cell, Volume *72*

Supplemental Information

Deregulated Expression of Mammalian lncRNA through Loss of SPT6 Induces R-Loop Formation, Replication Stress, and Cellular Senescence

Takayuki Nojima, Michael Tellier, Jonathan Foxwell, Claudia Ribeiro de Almeida, Sue Mei Tan-Wong, Somdutta Dhir, Gwendal Dujardin, Ashish Dhir, Shona Murphy, and Nick J. Proudfoot

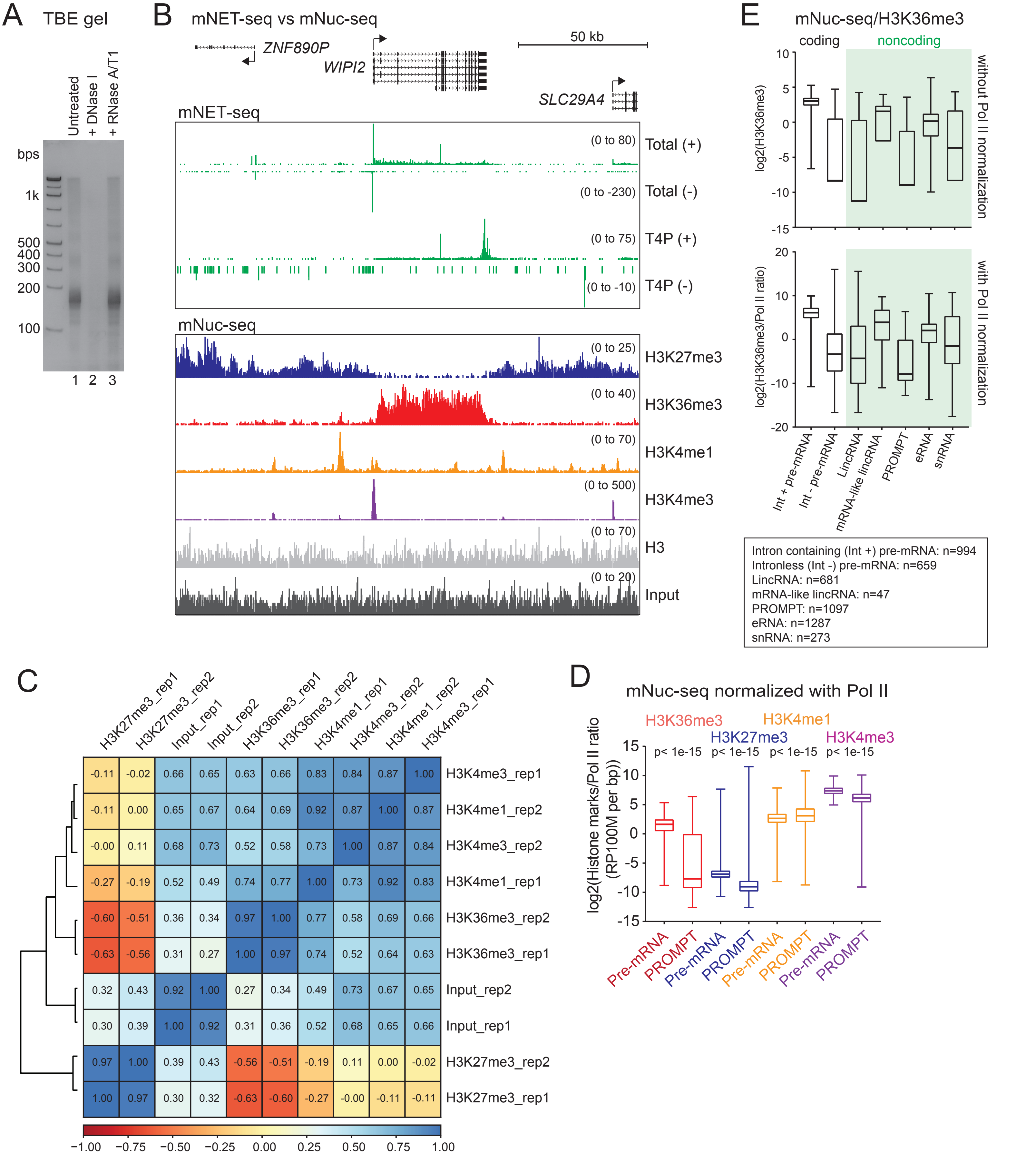

Figure S1. Establishment of mNuc-seq methodology. (related to Figure 1)
(A) Gel image of nucleosome pattern. Following MNase digestion, purified chromatin from untreated (lane 1), DNase I (lane 2) and RNase A/T1 mix (lane 3). Mononucleosome sized (160-180 bps) DNA is predominant.
(B) Wider view (<150 kb) of WIPI2 analysed by mNET-seq/Total and T4P. mNuc-seq/H3K36me3 and K27me3 marks correlate positively and negatively with mNET-seq signals respectively.
(C) Correlation matrix heatmap for mNuc-seq. Positive (+ blue): Negative (- red) showing negative correlation of H3K36me3 and H3K27me3.
(D) Box plots of indicated mNuc-seq signals normalized with mNET-seq/Total CTD signals on pre-mRNA and PROMPT regions.
(E) Box plots of mNuc-seq/H3K36me3 signals normalized without (top) and with (bottom) mNET-seq/Total signals on indicated gene classes.

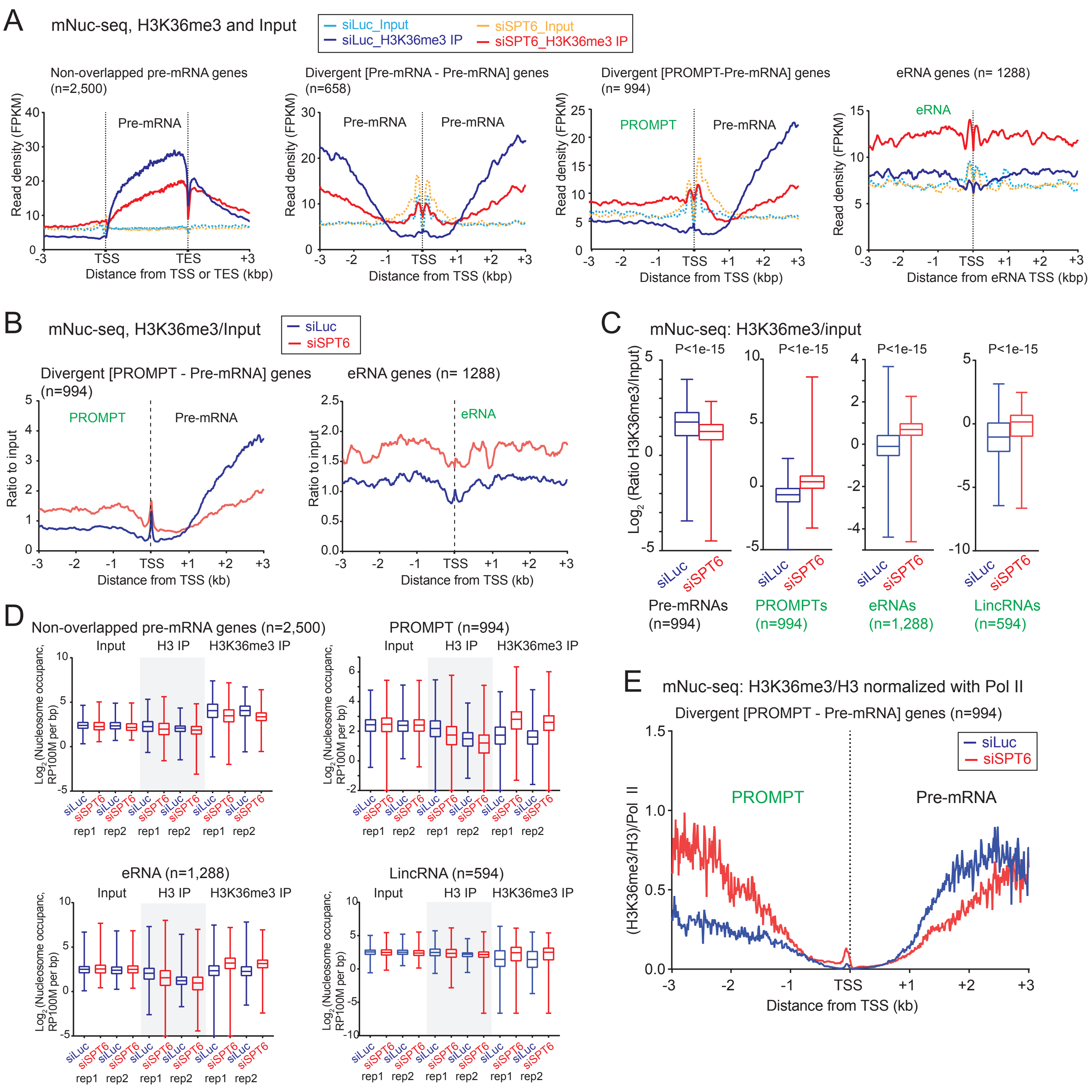

Figure S2. H3K36me3 distribution on pre-mRNA and lncRNA genes. (related to Figure 2)

(A) Meta-analysis of mNuc-seq/H3K36me3 signals over -/+3 kb regions of non-overlapped pre-mRNA genes (left), divergent TUs [Pre-mRNA–Pre-mRNA] (middle left), [PROMPT–Pre-mRNA pairs] (middle right) and enhancers [eRNA-eRNA] (right) upon SPT6 depletion. Dashed lines represent input signals.

(B) Meta-analysis of mNuc-seq/H3K36me3 signal ratioed to input over -/+ 3 kb regions of divergent TUs [PROMPT – Pre-mRNA] (left) and [eRNA -eRNA] (right).

(C) Box plots quantification of mNuc-seq/H3K36me3 ratioed to input on pre-mRNA genes (whole annotated gene), PROMPTs (3 kb from TSS), eRNA (2 kb from the center) and lincRNA (whole annotated gene).

(D) Box plots quantification of absolute signal intensity of mNuc-seq/input, H3 and H3K36me3 on non-overlapping pre-mRNA genes (whole annotated gene), PROMPT (3 kb from TSS), eRNA (2 kb from center) and lincRNA (whole annotated gene) regions. Two replicates are shown.

(E) Meta-analysis of mNuc-seq/H3K36me3 ratioed to H3 and also normalized with Total CTD mNET-seq level over -/+ 3 kb regions of divergent TUs [PROMPT - Pre-mRNA].

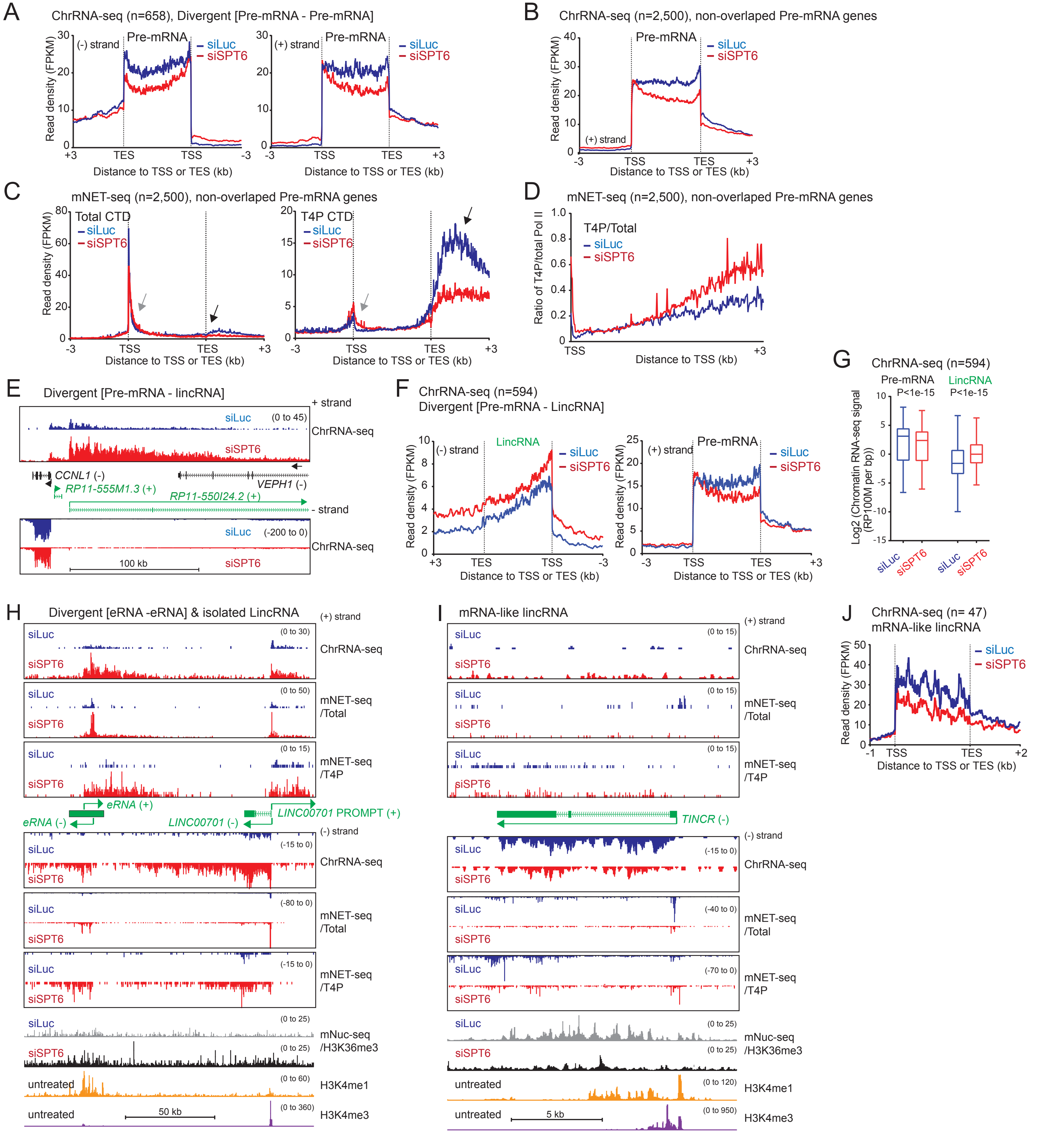

Figure S3. SPT6 depletion reduces Pol II elongation on pre-mRNA, while increasing transcription of lincRNA TUs. (related to Figure 3)
(A) Meta-analysis of reads density for strand-specific ChrRNA-seq signals from 3 kb TSS upstream to 3 kb TES downstream of divergent [Pre-mRNA-Pre-mRNA] genes.
(B) Meta-analysis of reads density for strand-specific ChrRNA-seq signals from 3 kb TSS upstream to 3 kb TES downstream of non-overlapped pre-mRNA genes.
(C) Meta-analysis of reads density for mNET-seq/Total CTD (left) and T4P (right) signals from 3 kb TSS upstream to 3 kb TES downstream of non-overlapped pre-mRNA genes.
(D) Meta-analysis of ratio of mNET-seq/T4P to total CTD from TSS to + 3 kb of non-overlapped pre-mRNA genes.
(E) *CCNL1* and *RP11-550124.2* as example of promoter-associated lincRNA affected by SPT6 depletion.
(F) Meta-analysis of reads density for strand-specific ChrRNA-seq signals from 3 kb TSS upstream to 3 kb TES downstream of divergent [LincRNA - Pre-mRNA] genes.
(G) Box plots of ChrRNA-seq signals (whole annotated gene) of Pre-mRNA and lincRNA pairs [LincRNA - Pre-mRNA].
(H) Examples of eRNA–eRNA pair and linked lincRNA, *LINC00701*.
(I) Example of mRNA-like lincRNA, *TINCR*. mNuc-seq/H3K4me3 and me1 are shown as marks of active promoters and enhancers, respectively.
ChrRNA-seq and mNET-seq profiles (+ and – strands) of *TINCR* show that SPT6 depletion causes transcription elongation defect as for pre-mRNA gene.
(J) Meta-analysis of reads density for strand-specific ChrRNA-seq signals from 3 kb TSS upstream to 3 kb TES downstream of mRNA-like lincRNA TUs upon SPT6 depletion.

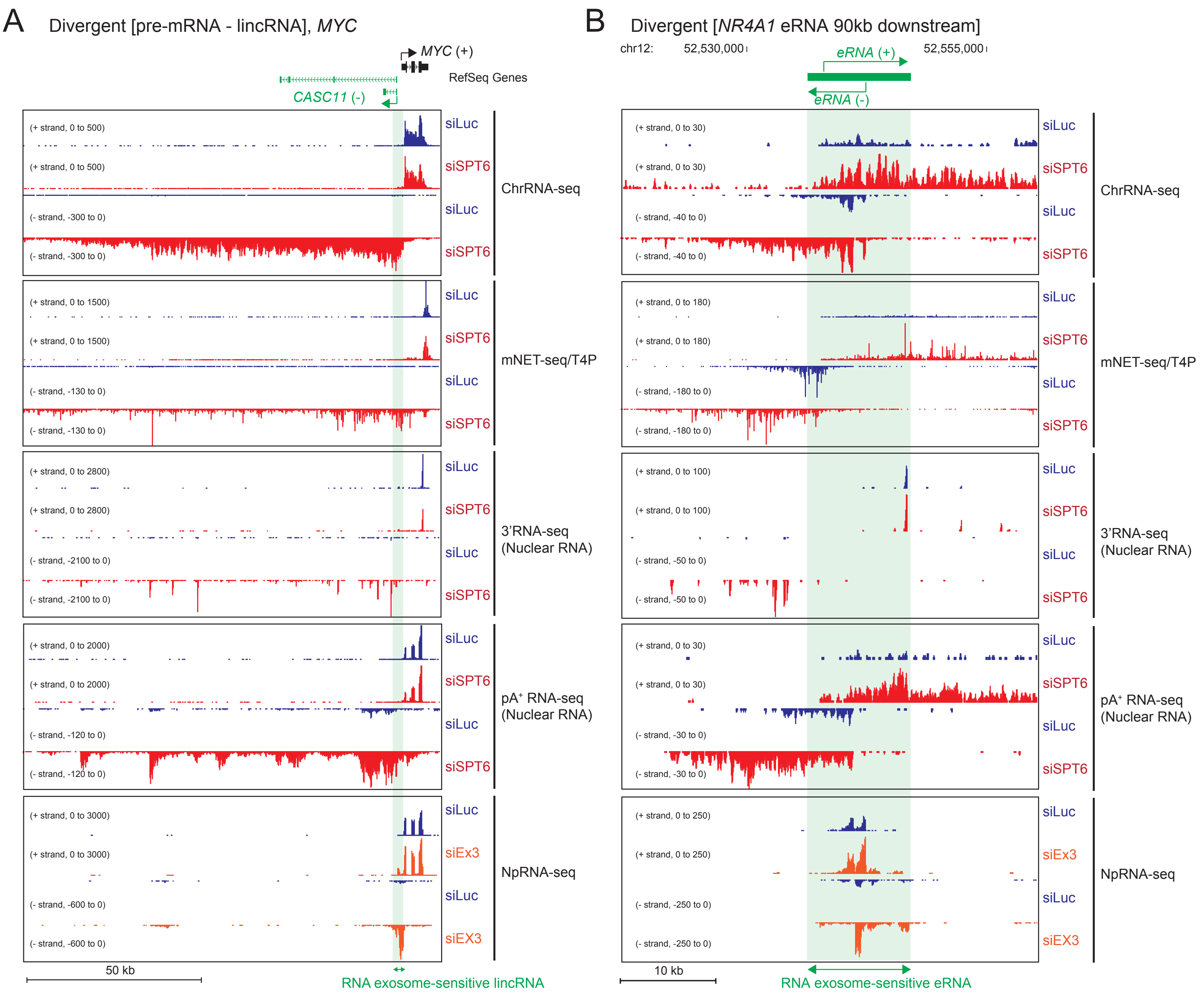

Figure S4. *CASC11* lincRNA - *MYC* pre-mRNA and *NR4A1* eRNA. (related to Figure 4)
(A) Divergent gene [*CASC11* lincRNA-*MYC* pre-mRNA] and (B) Divergent eRNA [*NR4A1* eRNA, 90kb downstream].
From top, strand-specific ChrRNA-seq, mNET-seq/T4P, 3' RNA-seq and pA+ RNA-seq from siLuc and siSPT6 transfected HeLa cells are shown.
NpRNA-seq from siLuc and siEX3 transfected HeLa cells are shown at bottom. RNA exosome-sensitive *MYC* PROMPT is highlighted in green.
SPT6 depletion caused overexpressed and extended lincRNA *CASC11* in chromatin. PAS usage (3'RNA-seq) and increased pA+ signals (pA+RNA-seq) were detected further downstream of highlighted PROMPT region. eRNA stabilised by exosome depletion highlighted in green. SPT6 depletion caused extended eRNA formation.

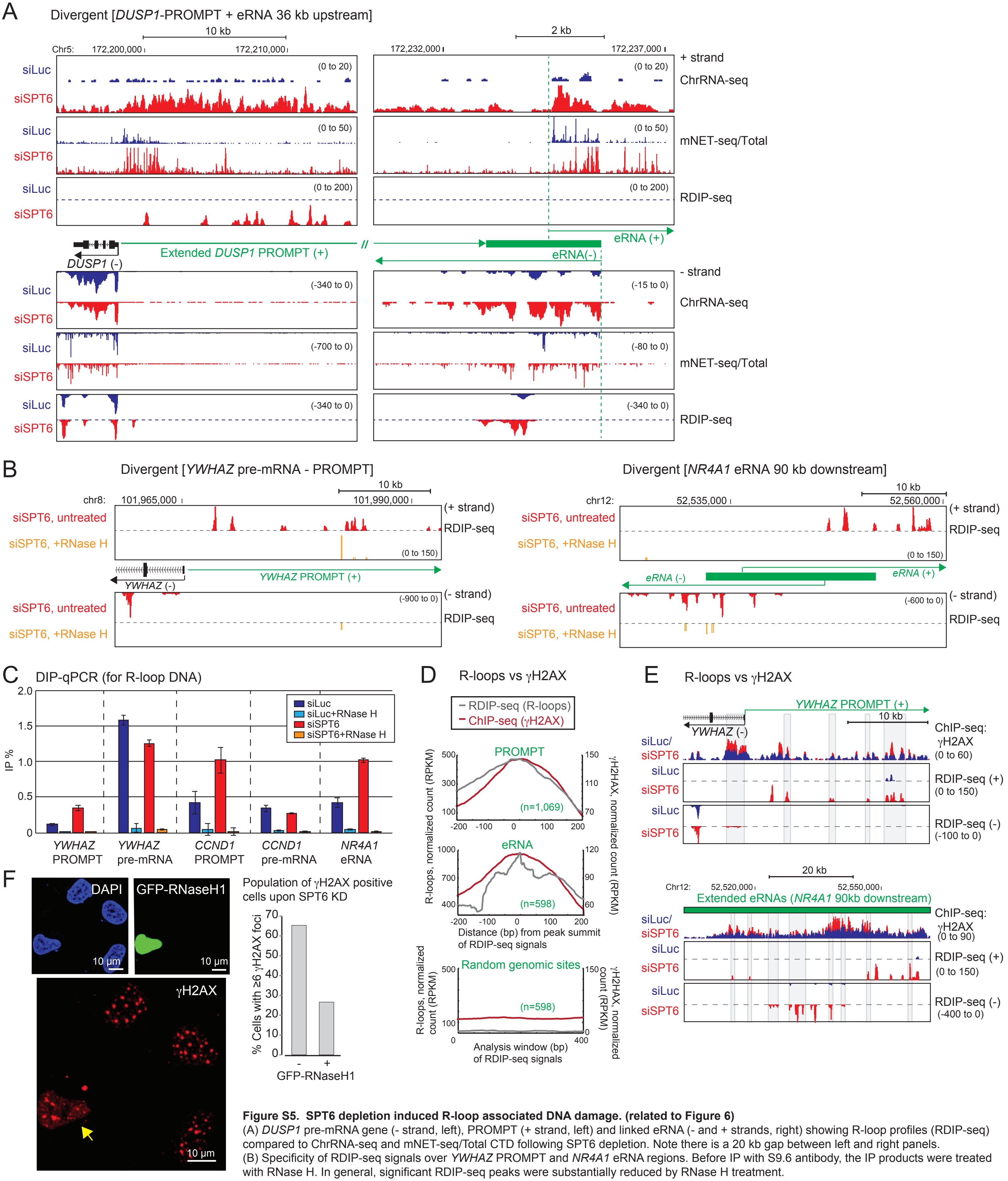

Figure S5. SPT6 depletion induced R-loop associated DNA damage. (related to Figure 6)
(A) *DUSP1* pre-mRNA gene (- strand, left), PROMPT (+ strand, left) and linked eRNA (- and + strands, right) showing R-loop profiles (RDIP-seq) compared to ChrRNA-seq and mNET-seq/Total CTD following SPT6 depletion. Note there is a 20 kb gap between left and right panels.
(B) Specificity of RDIP-seq signals over *YWHAZ* PROMPT and *NR4A1* eRNA regions. Before IP with S9.6 antibody, the IP products were treated with RNase H. In general, significant RDIP-seq peaks were substantially reduced by RNase H treatment.
(C) Quantitative DIP-PCR of indicated candidate gene loci. SPT6 depletion increased DIP signals in PROMPTs and eRNA, but decreased signal in pre-mRNA. In vitro RNase H treatment significantly reduced all DIP signals. Data is represented as mean ± SEM.
(D) Meta-analysis of RDIP-seq and γH2AX ChIP-seq in SPT6-depeleted HeLa cells for PROMPT and eRNA R-loop peak regions. Distance shown from RDIP-seq peak summit. Random genomic regions (400bp window) are shown as control.
(E) *YWHAZ* PROMPT and *NR4A1* eRNA are shown as examples of the peak summit analyses in (D). γH2AX ChIP-seq signals were overlapped in siLuc (blue) and siSPT6 (red) samples. R-loop peaks with increased γH2AX ChIP-seq signals are highlighted in grey.
(F) Immunofluorescence analysis of γH2AX after GFP-RNase H1 over-expression. A field of 4 cells is shown with 1 cell over-expressing RNase H1. γH2AX IF is 2-fold enlarged. Note that the RNase H1 over-expressing cell shows fewer foci. Cells which contains >6 nuclear foci of γH2AX were counted upon SPT6 knockdown.

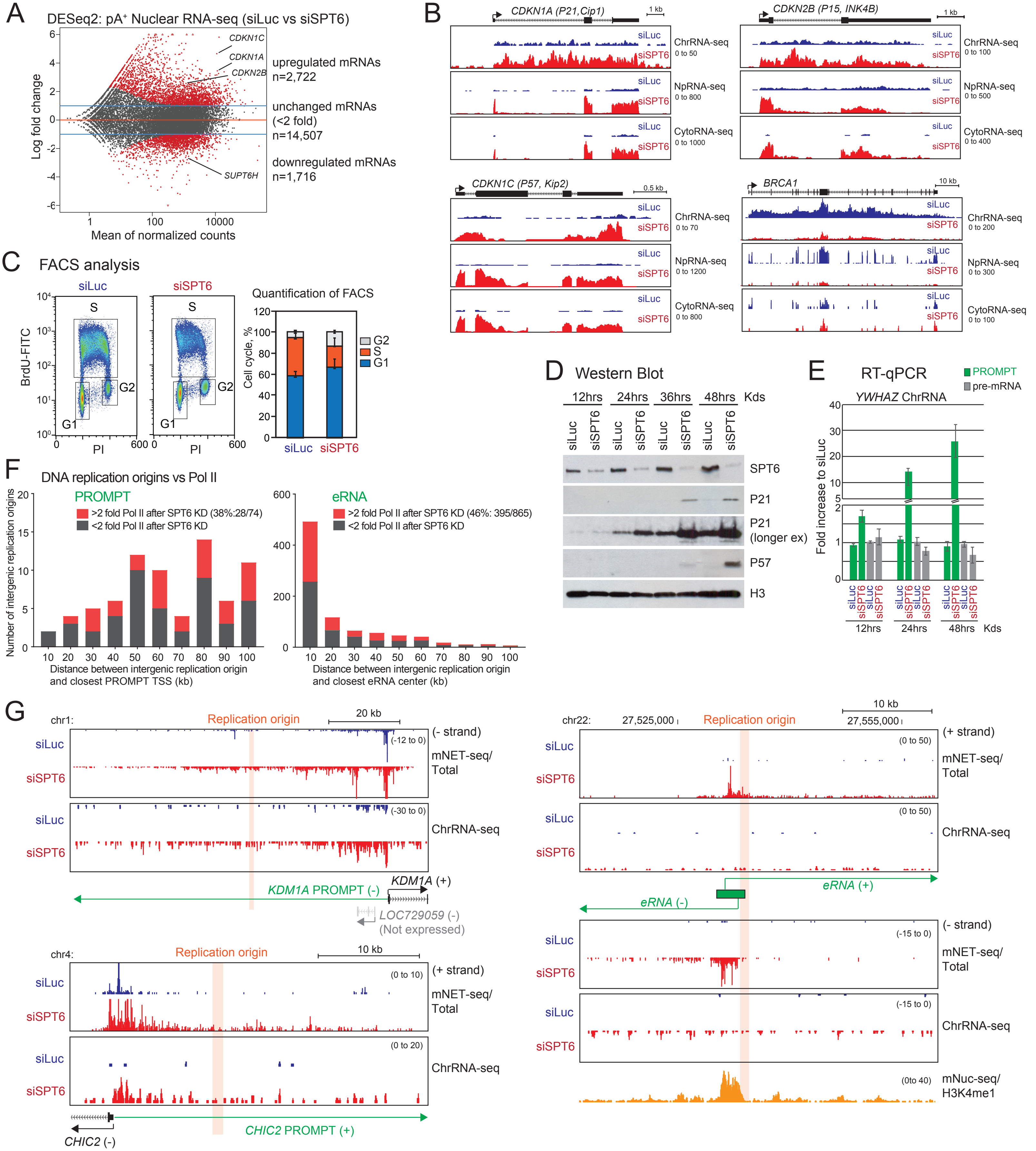

Figure S6. SPT6 depletion causes collision between Pol II and DNA replisome. (related to Figure 7)

(A) Differential expression-analysis (DESeq2) of nuclear pA+ RNA-seq signals from HeLa nucleus. >2-fold upregulated, > 2-fold downregulated and unchanged (<2-fold). Expression of cell cycle inhibitor genes such as *CDKN1A*, *CDKN1C* and *CDKN2B* were significantly upregulated while siRNA-target *SUPT6H* was downregulated.

(B) Examples of siSPT6-affected gene expression in different HeLa cell fractions: chromatin (ChrRNA-seq), nucleoplasm (NpRNA-seq) and cytoplasm (CytoRNA-seq). Signals of cell cycle inhibitor genes, *CDKN1A*, *CDKN1C* and *CDKN2B* were upregulated in all three fractions. In contrast DNA damage response gene *BRAC1* was down-regulated after SPT6 siRNA depletion.

(C) Cell cycle analysis by fluorescence activated cell sorting (FACS) of HeLa cells following SPT6 depletion. Signals were quantified from three biological replicates of SPT6-depleted HeLa cells. Data is represented as mean ± SEM.

(D) The whole HeLa cell extracts were prepared after 12, 24, 36 and 48 hr control and SPT6 siRNAs transfection for western blot against indicated antibodies. P21 levels are shown with short and long exposure. Note P21 protein level was upregulated after 24 hr SPT6 depletion.

(E) Quantitative RT-PCR of chromatin-bound RNA. *YWHAZ* PROMPT and pre-mRNA were analyzed after 12, 24 and 48 hr control and SPT6 siRNAs transfection. Data are represented as mean ± SEM.

(F) Spatial relationship between Pol II and DNA replication origin in intergenic region. Replication origins with elevated Pol II level (>2 fold) in SPT6 depleted cells are highlighted in red.

(G) Three examples of mNET-seq/Total profiles in intergenic DNA replication origins.
